# Origins and lifetimes of secular and tidal bars in simulated disc galaxies

**DOI:** 10.1093/mnras/stag428

**Published:** 2026-03-05

**Authors:** Matthew Frosst, Danail Obreschkow, Aaron Ludlow, A Fraser-McKelvie

**Affiliations:** International Centre for Radio Astronomy Research (ICRAR), University of Western Australia, Crawley, WA 6009, Australia; ARC Centre of Excellence for All Sky Astrophysics in 3 Dimensions (ASTRO 3D), Sydney, Australia; International Centre for Radio Astronomy Research (ICRAR), University of Western Australia, Crawley, WA 6009, Australia; ARC Centre of Excellence for All Sky Astrophysics in 3 Dimensions (ASTRO 3D), Sydney, Australia; International Centre for Radio Astronomy Research (ICRAR), University of Western Australia, Crawley, WA 6009, Australia; ARC Centre of Excellence for All Sky Astrophysics in 3 Dimensions (ASTRO 3D), Sydney, Australia; ARC Centre of Excellence for All Sky Astrophysics in 3 Dimensions (ASTRO 3D), Sydney, Australia; European Southern Observatory, Karl-Schwarzschild-Straße 2, D-85748 Garching, Germany

**Keywords:** instabilities, galaxies: bar, galaxies: evolution, galaxies: interactions

## Abstract

We analyse 307 Milky Way-mass disc galaxies in the TNG50 cosmological simulation to study the formation and evolution of stellar bars through secular processes and tidal interactions. About 90 per cent of these galaxies form at least one bar during their cosmic evolution. Most bars form rapidly in dynamically cold discs shortly after the central stellar mass exceeds that of dark matter (inside the stellar half-mass radius). In these cases, bar formation is consistent with secular instabilities driven by the disc’s self-gravity, which organizes stellar orbits into a coherent bar structure. However, about 24 per cent of barred galaxies are dark matter-dominated at the time of bar formation. We trace the origin of these bars to tidal perturbations from passing or accreting satellites and streams, and introduce a new metric, $\mathcal {S}_{\rm bar}$, to quantify the total external tidal field acting on each galaxy. We find that $\mathcal {S}_{\rm bar}$ correlates negatively with the central stellar-to-dark matter mass ratio at the time of bar formation: the more dark-matter-dominated a disc, the stronger the tidal perturbation required to trigger a bar. Bars that form in dark matter-dominated discs under tidal perturbations are typically transient – disappearing in a few Gyr – unlike their counterparts which form in stellar-dominated discs. After a few orbital times, the properties of all bars are broadly similar, though their host galaxies differ: secular bars arise in thin, compact discs, whereas tidally induced bars can form in thicker, more extended discs whose properties closely resemble those of unbarred galaxies.

## 1 INTRODUCTION

The recent explosion of observations of high-redshift galaxies has revealed an early Universe populated by settled, disc-like galaxies (L. Ferreira et al. 2023; C. J. Conselice et al. 2024). Stellar bars, too, are now regularly observed out to $z \approx 1{-}3$ (e.g. Y. Guo et al. 2023; Z. A. Le Conte et al. 2024; J. M. Espejo Salcedo et al. 2025; Y. Guo et al. 2025), indicating that bar formation is not restricted to low-*z* discs. They are believed to form via two processes: the secular growth of a global disc instability (F. Hohl 1971; G. Efstathiou, G. Lake & J. Negroponte 1982; M. S. Fujii et al. 2018; J. Bland-Hawthorn et al. 2024) and external tidal interactions that are common in cosmological environments (M. Noguchi 1987; I. Berentzen et al. 2004; E. L. Łokas et al. 2014). Despite their ubiquity, it remains uncertain whether bars that form secularly can be distinguished from those that form as a result of tidal interactions, particularly long after their formation (M. K. Cavanagh et al. 2022; E. J. Iles et al. 2024; Y. Zheng & J. Shen 2025).

Numerical simulations show that bars form secularly in dynamically cold, self-gravitating discs with high stellar-to-dark matter (DM) mass ratios (e.g. E. Athanassoula & J. A. Sellwood 1986; J. A. Sellwood 2014; M. S. Fujii et al. 2018; J. Bland-Hawthorn et al. 2023; M. Frosst, D. Obreschkow & A. Ludlow 2024), while DM-dominated galaxies may not form bars. Other disc properties, like thickness (e.g. A. Klypin et al. 2009; M. Aumer & J. Binney 2017; S. Ghosh et al. 2023), central velocity dispersion (e.g. J. A. Sellwood & R. G. Carlberg 1984; E. Athanassoula 2003), and gas fraction (I. Berentzen et al. 2004; F. Bournaud, F. Combes & B. Semelin 2005), can influence the onset and speed of secular bar growth. Once formed, secular bars tend to persist for many Gyr (e.g. E. Athanassoula 2013).

However, galaxies are subject to frequent interactions (F. Hammer et al. 2009). Simulations show that stellar bars can form rapidly in response to the associated tidal perturbations, particularly during mergers (e.g. M. Noguchi 1987; I. Berentzen et al. 2004) or flybys (e.g. M. Lang, K. Holley-Bockelmann & M. Sinha 2014; E. L. Łokas et al. 2014; E. L. Łokas 2018), during which strong tidal forces act on the disc (see also L. Mayer & J. Wadsley 2004; E. Romano-Díaz et al. 2008; I. Martinez-Valpuesta et al. 2017; T. Zana et al. 2018a; N. Peschken & E. L. Łokas 2019; Y. Rosas-Guevara et al. 2024). These perturbations can lead to bar formation in galaxies that would otherwise be stable to bar growth (M. Noguchi 1987; E. Romano-Díaz et al. 2008; M. Lang et al. 2014; Y. Zheng & J. Shen 2025). Furthermore, tidal bars may be short-lived and prone to destruction (N. Peschken & E. L. Łokas 2019). Cosmological simulations reveal a wide range of bar lifetimes, with some galaxies hosting long-lived bars (e.g. K. Kraljic, F. Bournaud & M. Martig 2012; C. Scannapieco & E. Athanassoula 2012; D. G. Algorry et al. 2017; N. Frankel et al. 2022; F. Fragkoudi et al. 2025), and others experiencing multiple episodes of bar formation, termination, and regeneration (I. Berentzen et al. 2004; E. Romano-Díaz et al. 2008; S. Ansar et al. 2025).

Whether tidally induced and secular bars have distinct characteristics is an open question. Early simulations by M. Noguchi (1987) suggest that tidal bars resemble those that formed secularly, exhibiting similar lengths, strengths, and pattern speeds (see also R. Moetazedian et al. 2017; T. Zana et al. 2018a). However, later studies reported that tidally induced bars are stronger (e.g. T. Miwa & M. Noguchi 1998; E. L. Łokas et al. 2014; E. L. Łokas 2018; N. Peschken & E. L. Łokas 2019) and rotate more slowly than their secular counterparts (I. Martinez-Valpuesta et al. 2017; A. R. Pettitt & J. W. Wadsley 2018). Y. Zheng & J. Shen (2025) reconciled this difference by using idealized simulations to show that slowly rotating tidal bars tend to form in galaxies that are otherwise stable against secular bar formation. In contrast, they show that tidal bars can also rotate as rapidly as secular bars, but only in galaxies that could *also* have formed the bar secularly (see also I. Martinez-Valpuesta et al. 2017). This again highlights the importance of disc properties in modulating the impact of tidal bar formation. Given what may be stark differences, is there a reliable way to distinguish between secular and tidal bars after they have formed?

Most previous studies comparing tidal and secular bars were based on idealized (e.g. M. Lang et al. 2014; I. Martinez-Valpuesta et al. 2017; E. L. Łokas 2018; E. J. Iles et al. 2024; Y. Zheng & J. Shen 2025) or zoom-in simulations (e.g. T. Zana et al. 2018a, b; S. Ansar et al. 2025). While these approaches allow for high spatial and mass resolution (and controlled environments in the case of idealized runs), they do not capture the diversity of galaxy–galaxy interactions that may affect the properties of tidal bars (A. Curir, P. Mazzei & G. Murante 2006). However, cosmological simulations of large volumes now reach sufficient resolution to reliably model the formation and evolution of stellar bars in statistically meaningful numbers of Milky Way-mass (MW) galaxies, the mass regime at which bars are most commonly found (T. Melvin et al. 2014; G. Gavazzi et al. 2015). This has enabled detailed studies of the origins of bars in their full cosmological context: for instance, Y. Rosas-Guevara et al. (2024) used the TNG50 simulation to study the transformation of disc galaxies between $z=0$ and 1, and found that roughly one-third of all bar formation events were tidally driven. P. D. López et al. (2024) studied a smaller sample of disc galaxies in TNG50 and found that while barred galaxies inhabit denser environments, it was challenging to link bar formation to previous mergers or flybys. In contrast, N. Peschken & E. L. Łokas (2019) analysed stellar bars in the illustris simulation, and found that nearly all were tidally induced, which they suggested was due to their low resolution and large softening lengths. The different conclusions of these studies expose the uncertainty surrounding environmental triggers of bar formation.

To address this uncertainty, we will analyse a sample of 307 $z=0$ disc galaxies with stellar masses comparable to that of the Milky Way, drawn from the TNG50 simulation. Galaxies in this mass range are well-studied in the context of bar formation (e.g. Y. Rosas-Guevara et al. 2022; S. Khoperskov et al. 2024; A. Pillepich et al. 2024; M. Semczuk et al. 2024; M. Frosst et al. 2025), and are sufficiently well resolved to robustly model bar evolution (see e.g. convergence studies of J. Dubinski, I. Berentzen & I. Shlosman 2009; M. Frosst et al. 2024). By following these galaxies from $z=4$ to 0, we will investigate how disc properties and tidal interactions influence stellar bar formation, and whether tidally induced and secular bars can be distinguished.

This paper is organized as follows: in Section 2, we describe the TNG50 simulation and the selection criteria for our galaxy sample. Section 3 details our methods for characterizing bar properties and quantifying the frequency of bar formation episodes. In Section 4, we introduce a metric to quantify the tidal fields acting on galaxies and use it to characterize galactic tides near the time of bar formation. In Section 5, we present our results on how galaxy and bar properties are related to the underlying formation mechanisms. We summarize our findings in Section 6.

## 2 METHODOLOGY

Throughout the paper, we define the stellar mass of a galaxy, ${\rm M}_{\star }$, as the total mass of stellar particles bounded by a $r=30\, {\rm kpc}$ sphere that is centred on the particle with the minimum potential energy, which we define as the galaxy centre. We adopt a cylindrical coordinate system coincident with the galaxy centre and align the *z*-axis with the net angular momentum vector of all stellar particles and star-forming gas cells within two stellar half mass radii, i.e. $2\times r_{\star ,1/2}$; we measure $r_{\star ,1/2}$ from the cumulative mass distribution of bound stellar particles within $r\le 30\, {\rm kpc}$. In this coordinate system, $r = (R^2 + z^2)^{1/2}$ denotes the three-dimensional radius, where $R=(x^2+y^2)^{1/2}$ is the distance from the *z*-axis.

### 2.1 The TNG50 simulation

TNG50-1 (TNG50 hereafter; A. Pillepich et al. 2018a; D. Nelson et al. 2019) is the highest resolution simulation of the TNG project (F. Marinacci et al. 2018; J. P. Naiman et al. 2018; D. Nelson et al. 2018; V. Springel et al. 2018). It was run using the arepo moving-mesh code (V. Springel 2010) with cosmological parameters taken from the Planck Collaboration XIII (2016) results.

TNG50 follows the evolution of DM, gas cells, and stellar and black hole particles in a 51.7 cubic cMpc volume from $z=127$ to $z=0$ (the prefix ‘c’ indicates co-moving Mpc). At the initial redshift, the simulation contains $2160^3$ fluid elements that have a target mass of $m_{\rm gas}=8.5\times 10^4\, {\rm M}_\odot$ and an equal number of DM particles of mass $m_{\rm DM}=4.5\times 10^5{\, {\rm M}_{\odot }}$. TNG50 employs sub-grid models for unresolved physics including heating and cooling, star formation and stellar evolution, chemical enrichment, supermassive black hole seeding and growth, and feedback from stars and active galactic nuclei (see F. Marinacci et al. 2018; J. P. Naiman et al. 2018; D. Nelson et al. 2018; V. Springel et al. 2018; A. Pillepich et al. 2018b, for details). The sub-grid model parameters were calibrated as described in R. Weinberger et al. (2017).

The gravitational softening for gas cells is dynamically adapted to the effective cell size, but reaches a minimum physical value of $\epsilon _{\rm gas} = 72$ pc. The softening length for collisionless (DM and stellar) particles is $\epsilon _{c} = 575$ cpc until $z=1$, and is fixed at $\epsilon _{c} = 288$ pc at lower redshifts.

Galaxies and DM haloes were identified using the subfind algorithm (V. Springel et al. 2001; K. Dolag et al. 2009) and linked between consecutive snapshots using the sublink merger tree code (V. Rodriguez-Gomez et al. 2015); this also returns the virial properties of the halo, for instance, $M_{\rm 200c}$, the mass within the radius $r_{\rm 200c}$ that encloses a mean density equal to $200\times$ the critical density of the universe, $\rho _{\rm crit}$.

### 2.2 The disc galaxy sample

We focus our analysis on a sample of well-resolved galaxies that host prominent discs at $z=0$. We characterize the morphologies of galaxies using $\kappa _\star$, i.e. the fraction of stellar kinetic energy in ordered rotation, defined by L. V. Sales et al. (2010) as


(1) \begin{eqnarray*}
\kappa _{\star } = \frac{\sum _{k} m_{k}(j_{z,k}/R_{k})^{2}}{\sum _{k}m_{k}v_{k}^{2}},
\end{eqnarray*}

where $j_{z,k}$, $v_{k}$, and $m_{k}$ are the *z*-component of the specific angular momentum, velocity magnitude, and mass of the $k^{\rm th}$ particle, respectively, and the sum extends over all stellar particles with $r_k\le 3r_{\star ,1/2}$. Galaxies with prominent stellar discs have higher values of $\kappa _{\star }$ than dispersion-dominated spheroids (e.g. C. A. Correa et al. 2017; V. Rodriguez-Gomez et al. 2017).

The sample of disc galaxies used in our analysis includes all 307 disc galaxies in TNG50 that satisfy the following criteria at $z = 0$:

- A stellar mass, $M{\star }$, in the range $10^{10.5}{\rm M{\odot }} < M{\star } \le 10^{11.2}\, {\rm M{\odot }}$;
-
$\kappa _{\star } \ge 0.3$ ;
- No other galaxy with $M_{\star } \ge 10^{10}\, {{\rm M}_{\odot }}$ lies within 100 kpc of their centres.

This sample includes the 198 MW/M31 analogues curated by A. Pillepich et al. (2024), and extends it to a broader range of environments. Criterion (iii) excludes galaxies that are likely undergoing major mergers or are within cluster environments at $z=0$.

## 3 BAR FORMATION AND EVOLUTION

### 3.1 Characterizing the formation of stellar bars

We determine the presence of stellar bars using a Fourier decomposition of the two-dimensional, face-on stellar surface mass density of each disc (E. Athanassoula 2002; R. Guo et al. 2019; W. Dehnen, M. Semczuk & R. Schönrich 2023; M. Frosst et al. 2024). Specifically, we focus on the $m=2$ cylindrical mode, defined as


(2) \begin{eqnarray*}
\mathcal {A_{\rm 2}} = \frac{\sum _k m_{k} e^{2i\theta _{k}}}{\sum _k m_{k}},
\end{eqnarray*}

where $m_{k}$ and $\theta _{k}$ are the mass and azimuthal angle of the $k^{\rm th}$ star particle. We use this to construct radial profiles of the bar strength,


(3) \begin{eqnarray*}
A_{\rm 2}(R) =|\mathcal {A}_{\rm 2}(R)|,
\end{eqnarray*}

and its position angle,


(4) \begin{eqnarray*}
\phi _2(R)=\frac{1}{2}\arg (\mathcal {A}_2(R)).
\end{eqnarray*}

The $A_{2}(R)$ and $\phi _{2}(R)$ profiles are calculated in cylindrical shells that contain $N_{\rm part}=10^4$ stellar particles. From these two profiles, we define four bar properties:

- The characteristic bar strength, $A_{2}^{\rm max}$, defined at the radius where $A_{2}(R)$ reaches a maximum.
- The bar position angle, $\phi _{2}^{\rm max}$, defined as the value of $\phi _{2(R)}$ at the same radius at which $A_{2}(R)$ reaches a maximum.
- The bar length, $R_{\rm bar}$, defined as the radius of the largest shell where $A_{2}(R_{\rm bar}) \ge A_{2}^{\rm max}/2$ and $|\phi _{2}(R_{\rm bar})| \le \phi _{2}^{\rm max} + 10^{\circ }$.
- The instantaneous bar pattern speed, $\Omega _{\rm bar}$, is calculated from the time derivative of $\phi _{2}^{\rm max}$ while accounting for particle flux between bins (following the method outlined by W. Dehnen et al. 2023).

Further details on our measurements of bar properties can be found in Appendix A.

Following Y. Rosas-Guevara et al. (2022), a galaxy is considered barred when $A_{2}^{\rm max} \ge 0.2$ and $R_{\rm bar} \ge 1.4 \, \epsilon _{\rm c}$ are simultaneously satisfied and persist for at least three consecutive snapshots ($\sim 450\, {\rm Myr}$ for TNG50). While somewhat arbitrary, these choices result in a sample of barred galaxies, which we confirmed by visual inspection. With this, we measure the bar formation time, $t_{\rm bar}$, as the start of a new bar episode, and defined as the first snapshot at which a galaxy is considered barred. We consider an existing bar episode to end if $A_{2}^{\rm max} < 0.2$ for at least three consecutive snapshots; the bar termination time corresponds to the last snapshot before this criterion is satisfied. For the termination of bars, we do not consider $\phi _2$ or $R_{\rm bar}$, as these quantities are sensitive to fluctuations induced by flybys with passing satellite galaxies, even during interactions where stellar bars are visually present. Throughout a galaxy’s evolution there may be several bar episodes, and thus several formation and termination times. The bar lifetime is simply the time interval between $t_{\rm bar}$ and its termination time (or $z=0$, in which case it is a lower limit).

### 3.2 Episodes of bar formation in galactic discs

**Figure 1.**
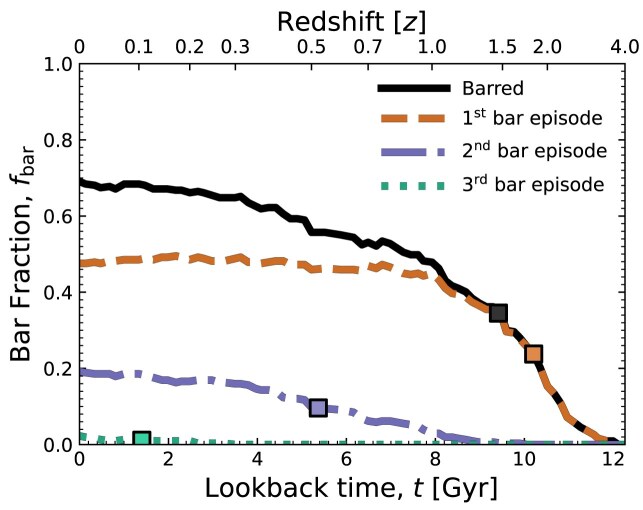
Evolution of the instantaneous bar fraction, $f_{\rm bar}$, for the full galaxy sample (black). The brown, purple, and green lines show the fraction of galaxies currently in their first, second, or third bar episode, respectively. Coloured squares mark the time at which these lines reach half their $z=0$ value.

The solid black line in the top panel of Fig. 1 shows the instantaneous fraction of barred galaxies, $f_{\rm bar}$, as a function of time, *t*. The brown, purple, and green lines show the fractions of galaxies on their first, second, or third bar episode, respectively. These coloured lines add up to the total bar fraction (black line) at any time. Coloured squares along the lines mark the time at which each population reaches half its $z=0$ value.

The instantaneous $f_{\rm bar}$ in Fig. 1 increases from about 2 per cent at $z=4$ to 69 per cent at $z=0$ (corresponding to 212 barred discs). This is consistent with other studies based on MW-mass disc galaxies in TNG50 (I. D. Gargiulo et al. 2022; T. Zana et al. 2022; M. Frosst et al. 2025) and with the analysis of other simulations that used similar galaxy selection criteria (F. Fragkoudi et al. 2025). However, our bar fraction is higher than that obtained by Y. Rosas-Guevara et al. (2022), which likely reflects their inclusion of lower-mass disc galaxies, where bars are less common in TNG50 (e.g. M. Roshan et al. 2021). Of the galaxies in our sample that are barred at $z=0$, we find that 69 per cent have experienced only one bar episode, with that bar persisting to the end of the simulation. Another 28 per cent are in their second bar episode, half of which have formed by $z \approx 0.5$. Only 3 per cent have had three bar episodes, half of which have formed by $z \approx 0.15$.

Regardless of whether the bars survive to $z=0$, when considering the full history of bars formed within our sample of discs, we find that 66 per cent of galaxies undergo only one bar episode (203 galaxies), 24 per cent experience multiple episodes (75 galaxies), and the remaining 10 per cent (29 galaxies) never form a bar. Among the galaxies with multiple episodes, 89 per cent form bars twice, and no galaxy experiences more than three. This upper bound may be due to the time resolution of our analysis and the relatively conservative criteria used to identify bar episodes.

Together, these results demonstrate that, while bar formation is common in massive discs, bar histories are diverse, and that a significant population of galaxies experience only a single, sustained bar throughout their evolution. While difficult to verify observationally, this appears to be in agreement with the large number of old bars derived from age dating nuclear discs (C. Sá-Freitas et al. 2025). For simplicity, we will focus hereafter on galaxies with only one bar episode. More complicated circumstances are left for future work.

### 3.3 The central stellar dominance of bar-forming galaxies

**Figure 2.**
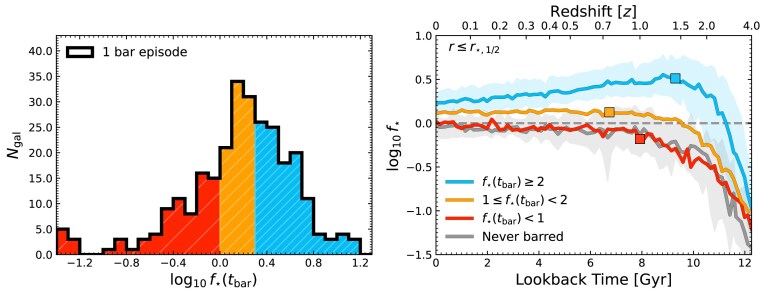
The left panel shows the distribution of the central stellar-to-DM mass ratio measured at the time of bar formation, $f_{\star }(t_{\rm bar})$, for all galaxies in our sample that form only one bar episode. Background colours indicate the $f_{\star }(t_{\rm bar})$ bins to be used throughout this work. In the right panel, we show the evolution of $f_{\star }$ from $z=4$ to $z=0$ for each $f_{\star }(t_{\rm bar})$ bin. Coloured squares indicate the median $t_{\rm bar}$ of these bins. The grey line indicates the evolution of galaxies that never form bars. Shaded regions show the interquartile range (IQR) for select $f_{\star }(t_{\rm bar})$ bins, but are representative of all others.

M. S. Fujii et al. (2018) used idealized simulations to show that secular bar formation occurs more rapidly in galaxies with higher disc-to-total mass fractions (see also F. Combes & R. H. Sanders 1981; E. Athanassoula & J. A. Sellwood 1986; J. Bland-Hawthorn et al. 2023). Motivated by this, in Fig. 2 we plot the time evolution of the central stellar-to-DM mass ratio, $f_{\star } = M_{\star }/M_{\rm DM}$, measured within $r\le r_{\star ,1/2}$, for galaxies in our sample (i.e. both masses are now measured within this radius). In principle, galaxies with high $f_{\star }$ should be able to rapidly form secular bars, but galaxies with low $f_{\star }$ should be comparatively stable against secular bar formation.

In the left panel of Fig. 2, we show as a thick black histogram the distribution of $f_{\star }$, at the time of bar formation, $t_{\rm bar}$, for all galaxies that form only one bar. The differently coloured regions under this line indicate whether these galaxies form their bars while highly centrally stellar-dominated [$f_{\star }(t_{\rm bar}) \ge 2$, blue, tightly hatched], marginally centrally stellar-dominated [$1\le f_{\star }(t_{\rm bar}) < 2$, orange hatched], or while centrally DM-dominated [$f_{\star }(t_{\rm bar}) < 1$, red, sparsely hatched], respectively. While many galaxies are stellar-dominated at the time of bar formation (76 per cent), as expected for secular bar formation, $\approx 24$ per cent are DM-dominated. Unlike stellar-dominated galaxies, DM-dominated systems are less susceptible to rapid secular bar formation and likely require external forces to trigger bar growth, as we explore in Section 4.

The right panel of Fig. 2 shows the redshift evolution of $f_{\star }$ for the galaxies split by $f_{\star }(t_{\rm bar})$ as in the left panel, with the same colours. Discs that never form bars are shown as a thick grey line. Coloured squares mark the median $t_{\rm bar}$ for each population, and the dashed horizontal grey line indicates $f_{\star } = 1$. Looking at the evolution of the galaxies in the different $f_{\star }(t_{\rm bar})$ bins, we see a few key trends: galaxies that form bars when highly stellar-dominated (blue line) experience the most rapid growth in $f_{\star }$ at early times, and form their bars on average only $\approx 2\, {\rm Gyr}$ after their discs become self-gravitating (half do so by $z\approx 1.5$), as expected for secular bar formation (see D. Izquierdo-Villalba et al. 2022; Y. Rosas-Guevara et al. 2022; S. Khoperskov et al. 2024; F. Fragkoudi et al. 2025; M. Frosst et al. 2025). However, as $f_{\star }(t_{\rm bar})$ decreases, bars form later, and $f_{\star }$ grows more slowly and peaks at lower values. The galaxies that form their bars while still DM-dominated (red line) tend to show the slowest growth in $f_{\star }$ over time, and are comparable to unbarred discs. Many of these galaxies never become stellar-dominated, but when they do it is far after $t_{\rm bar}$, and only at lower redshifts ($z \lesssim 1$). The similarities between the DM-dominated barred galaxies and those that do not form bars suggest that other factors are influencing bar formation.

### 3.4 Visual illustration of two barred galaxies

**Figure 3.**
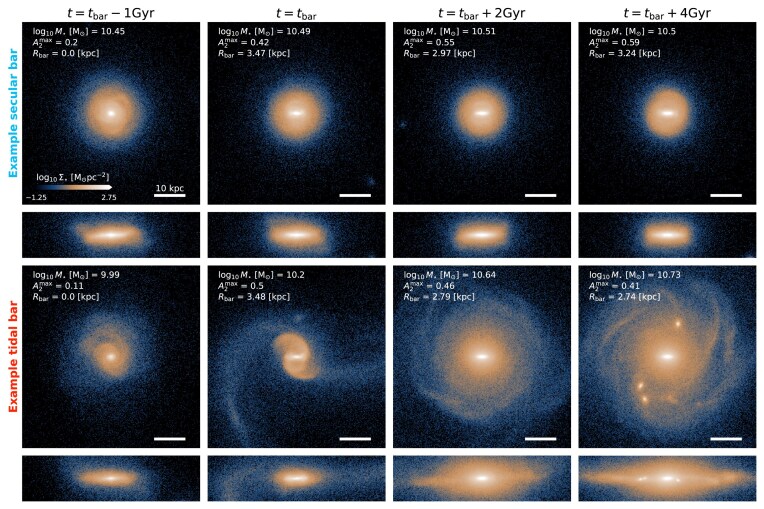
Face-on and edge-on stellar surface mass densities ($\Sigma _{\star }$) of two galaxies that form stellar bars while their discs are either stellar- (top) or DM-dominated (bottom). Their $z=0$  subfind IDs are 414 918 and 388544, respectively. From left to right, the panels show these galaxies 1 Gyr before $t_{\rm bar}$, at $t_{\rm bar}$, and 2 and 4 Gyr after $t_{\rm bar}$, respectively. In the top left corner of face-on panels, we plot the stellar mass, $M_{\star }$ (measured within $r \le 30$ kpc), the bar strength, $A_{\rm 2}^{\rm max}$, and bar length, $R_{\rm bar}$. Projections are coloured according to the stellar surface mass density. White lines in the bottom right of each panel illustrate the size of $10\, {\rm kpc}$.

Fig. 3 shows the evolution of the face-on and edge-on stellar surface mass density projections of two example galaxies (time moving from left to right). In the top row, the bar forms when stellar-dominated [$f_{\star }(t_{\rm bar}) \approx 3.2$], while in the bottom row the bar formed when DM-dominated [$f_{\star }(t_{\rm bar}) \approx 0.1$]. While both example galaxies form their bars rapidly, it is clear that they do so for different reasons. The stellar-dominated galaxy forms its bar in isolation, suggesting a secular origin. Conversely, the DM-dominated galaxy in the bottom row forms its bar during the close passage of a nearby satellite, characteristic of tidally induced bar formation.

Note also that the bars themselves are not easily distinguishable, with roughly similar strengths and lengths, though the secular bar is longer and stronger than the tidal bar after $4\, {\rm Gyr}$. However, the discs of these two example galaxies *are* visually distinct. For instance, after $t_{\rm bar}$, the galaxy that forms the secular bar is thinner than galaxy that forms the tidal bar, which is visually thicker and displays warps, spiral arms, and other non-axisymmetric extended stellar features, including diffuse light. Clearly, bars form in both stellar- and DM-dominated disc galaxies, and these systems may form bars through different formation mechanisms. To proceed, we require a reliable method to characterize the tidal fields acting on bar-forming galaxies.

## 4 TIDAL FIELDS NEAR BAR-FORMING GALAXIES

To some degree, all galaxies constantly evolve within external tidal fields that can influence a range of structural features, including disc warps, flares, and stellar bars (e.g. B. Moore, G. Lake & N. Katz 1998; O. Y. Gnedin 2003; S. H. Oh et al. 2008; E. Romano-Díaz et al. 2008; A. R. Pettitt & J. W. Wadsley 2018). These tidal asymmetries are often attributed to nearby satellite galaxies and are commonly modelled by treating satellites as point-mass perturbers acting on an otherwise axisymmetric disc potential. This approach has been widely applied in both observational (e.g. D. M. Elmegreen et al. 1991; I. D. Karachentsev & D. I. Makarov 1999; D. R. Weisz et al. 2011; S. Pearson et al. 2016) and simulation-based studies (e.g. I. Berentzen et al. 2004; E. L. Łokas et al. 2016; I. Martinez-Valpuesta et al. 2017; M. Semczuk, E. L. Łokas & A. del Pino 2017; N. Peschken & E. L. Łokas 2019; P. D. López et al. 2024; S. Ansar et al. 2025). However, such metrics oversimplify the complex environments around galaxies and treat satellites independently, often assuming the tidal field is associated with a single satellite that contributes the largest tidal perturbation. In reality, tidal fields arise from complex distributions of stars, gas, and DM, which can interact to either amplify or suppress tidal effects associated with a single passing satellite. Motivated by this, we construct a general estimator of the total tidal field acting on galaxies due to the full surrounding matter distribution.

### 4.1 Quantifying tidal perturbations

Our method for quantifying external tidal effects generalizes a standard metric used for single point-mass perturbers (e.g. P. D. López et al. 2024; S. Ansar et al. 2025; and similar to D. M. Elmegreen et al. 1991 or I. D. Karachentsev & D. I. Makarov 1999) to arbitrarily complex environments.

Quantifying external tidal perturbations prerequisites a delineation between the system (i.e. the galaxy) and the environment inflicting this perturbation. We follow the common approach defining the boundary between system and environment as a sphere centred on the galaxy, choosing $r_{\rm t} \equiv 4\, r_{\star ,1/2}$ as its radius.^1^

We quantify the external tidal perturbation via the environment-induced tidal acceleration field, that is the excess acceleration field relative to the system’s centre-of-mass acceleration. On the spherical boundary, this tidal acceleration $\boldsymbol {a}_{\rm t}$, normalized by the system’s own mean radial acceleration, is given by (derivation in Appendix B)


(5) \begin{eqnarray*}
\boldsymbol {a}_{\rm t} = \mathbb {T}\, \hat{\boldsymbol {p}},
\end{eqnarray*}

where $\boldsymbol {p}$ is the position vector (measured from the centre of the sphere) – and $\hat{\boldsymbol {p}}=\boldsymbol {p}/r_{\rm t}$ its unit-vector – of a point on the spherical boundary. The tensor $\mathbb {T}$ is the dimensionless tidal tensor,


(6) \begin{eqnarray*}
\mathbb {T} = \frac{r_{\rm t}^3}{M_{\rm t}}\sum _{k\in \rm {env}}\frac{m_{k}}{r_{k}^3} \left[3\, \frac{\boldsymbol {r}_k \otimes \boldsymbol {r}_k}{r_k^2}-\mathbb {I} \right],
\end{eqnarray*}

where $M_{\rm t}$ is the system mass (i.e. the total mass of all particles of any species within $r_{\rm t}$), $m_k$ and $\boldsymbol {r}_k$ are the masses and positions of particles in the environment (i.e. all particles of any species with $r_{k} > r_{\rm t}$), $\boldsymbol {r}_{k}\otimes \boldsymbol {r}_{k}$ is the outer product of $\boldsymbol {r}_{k}$ with itself, and $\mathbb {I}$ is the identity matrix (details in Appendix B).

In the case of a single external perturber of mass *m* at position $\boldsymbol {r}$, the tensor $\mathbb {T}$ simplifies to a product of a dimensionless scalar $\mathcal {T} = (m\, r_{\rm t}^3)/(M_{\rm t}\, r^3)$ and a trace-free symmetric matrix with eigenvalues $(+2, -1, -1)$. The eigenvector associated with the positive eigenvalue points along $\boldsymbol {r}$, where the tidal field causes a symmetric stretching, while inducing a uniform compression in the orthogonal plane. The scalar $\mathcal {T}$ is identical to the standard ‘scaled tidal index’ often used in the literature (e.g. P. D. López et al. 2024; S. Ansar et al. 2025; and similar to D. M. Elmegreen et al. 1991 or I. D. Karachentsev & D. I. Makarov 1999); hence $\mathbb {T}$ is a consistent generalization of this index to arbitrarily complex environments. In general, the eigenvalues of $\mathbb {T}$ are all different; however, the tensor remains always trace-free, meaning that, on average, the tidal stretching and compression cancel out (i.e. no volumetric change) – a key feature of tidal fields at liner order.

For our further analysis it is convenient to reduce the tensor $\mathbb {T}$ to a scalar metric $\mathcal {S}$. Considering the trace-free nature of $\mathbb {T}$, the most straightforward way of defining such a scalar is the Frobenius norm,


(7) \begin{eqnarray*}
\mathcal {S} = \sqrt{{\rm Tr}\left(\mathbb {T}^2\right)} = \sqrt{\textstyle \sum _{j=1}^3 \lambda _{j}^2}.
\end{eqnarray*}

For a single point mass perturber, $\mathcal {S}$ is identical to the familiar scaled tidal index $\mathcal {T}$ up to a constant factor $\sqrt{6}$. Our definition of $\mathcal {S}$ ignores the alignment between the tidal tensor and the galaxy. In principle, we could have made a different choice, such as projecting $\mathbb {T}$ on to the galactic plane. However, no statistically significant differences were found between $\mathcal {S}_{\rm bar}$ of equation (7) and projected alternatives, and we therefore use this simpler version. Our method is quantitively similar to O. Y. Gnedin (2003), but our implementation is quite different due to our smaller scales and superior resolution.

**Figure 4.**
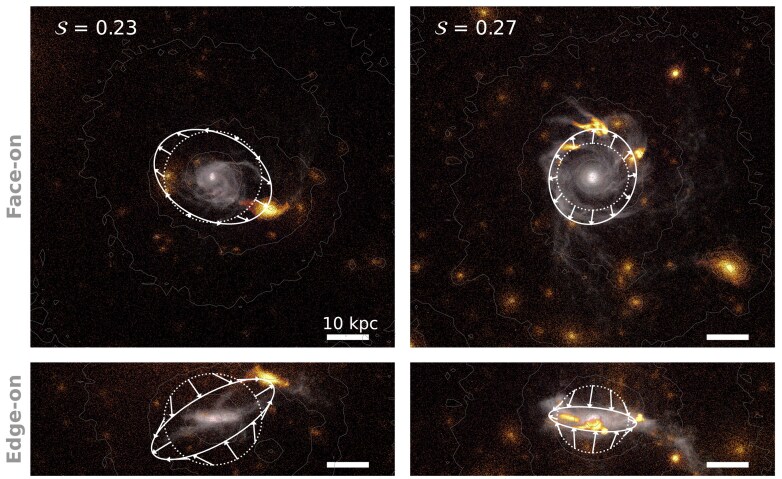
Face-on (top panels) and edge-on (bottom panels) surface mass densities of two example galaxies at $z=0.76$, when $\mathcal {S}$ reaches a maximum prior to the formation of a stellar bar (the $z=0$  subfind IDs are 441 709 and 432 106 for the galaxies in the left- and right-hand panel, respectively). The grey colour map corresponds to baryonic particles and cells (stars and gas) bound to the central galaxy whereas orange coloured points show those bound to satellite galaxies, but including the DM. White contours show the total DM density distribution. The dotted white circle in each panel indicates the radius $r_{\rm t} = 4\, r_{\star , 1/2}$ at which we measure the tidal field, $\mathcal {S}$. The white ellipse and associated arrows show how these circles are distorted due to each galaxy’s asymmetric tidal field (the magnitude of $\mathcal {S}$ is provided in the upper left corner of each panel). While satellite galaxies typically dominate the local tidal field, their collective influence must be considered to fully characterize the tidal perturbations experienced by galactic discs.

Fig. 4 shows the face-on and edge-on surface mass densities of two example galaxies at $z = 0.76$. Grey denotes all baryonic particles and cells bound to the central galaxy, while orange coloured regions indicate all material associated with satellite galaxies (including DM). Both systems are centrally DM-dominated yet develop stellar bars within one halo dynamical time, $t_{\rm dyn}$, of when they are shown. We define $t_{\rm dyn}$ as the redshift-dependent halo free-fall time,


(8) \begin{eqnarray*}
t_{\rm dyn} = \left(\frac{3\, \pi }{32\, G\, (200\, \rho _{\rm crit})}\right)^{1/2}.
\end{eqnarray*}

At $z=0$, $t_{\rm dyn} \approx 1.6\, {\rm Gyr}$, while at $z=2$, $t_{\rm dyn} \approx 0.5\, {\rm Gyr}$. One galaxy (left column) is accompanied by a single massive satellite and several less massive ones; the other (right column) is surrounded by multiple satellites of comparable mass. The dotted white sphere in each panel indicates $r_{\rm t}$. We compute $\mathcal {S}$ by summing over all collisionless particles and gas cells within $r_{\rm t} \le r_k \le 400\, \mathrm{kpc}$ of the galaxy centre,^2^ regardless of whether they are gravitationally bound. Despite differing satellite distributions, the two galaxies exhibit similar values of $\mathcal {S}$ (top-left corner of each face-on panel).^3^

Solid white ellipses and arrows in Fig. 4 illustrate how asymmetric tidal fields from nearby satellites cause accelerations normal to the face of the dotted spheres, with each ellipse having the same volume as its corresponding sphere. In the left column, the tidal field clearly points towards the dominant satellite and compresses it orthogonally. As this field is dominated by a single satellite, $\mathbb {T}$ has only one positive eigenvalue. In the right column, the tidal field is more complex: it is stretched in the plane of the disc towards a triplet of satellites (just above the dotted circle in the top right panel) and compressed perpendicularly to them. In this case, $\mathbb {T}$ has *two* positive eigenvalues, a situation not possible in the point-mass assumption. This shows that $\mathcal {S}$ can flexibly capture the deformation of the tidal field associated with a variety of environments.

### 4.2 Secular versus tidal bars

**Figure 5.**
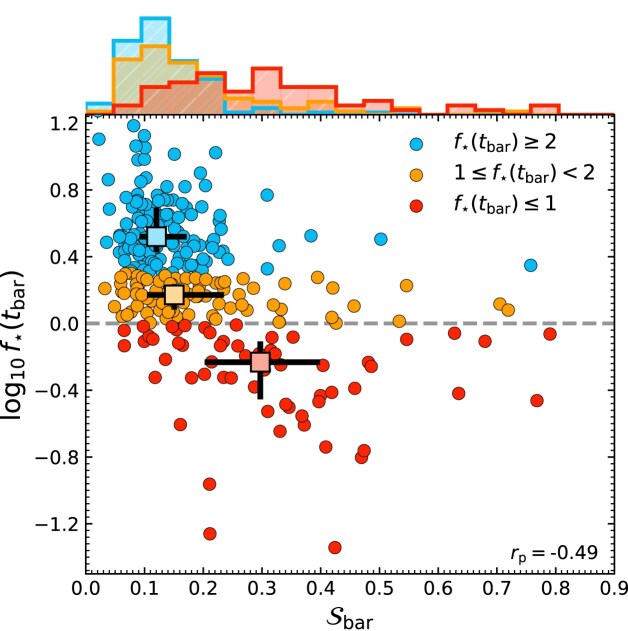
The relationship between central stellar-to-DM mass ratio at the time of bar formation, $f_{\star }(t_{\rm bar})$, and maximum tidal strength before bar formation, $\mathcal {S}_{\rm bar}$, for galaxies with 1 bar episode. Points are coloured according to $f_{\star }(t_{\rm bar})$, as in Fig. 2. The light-coloured squares in the main panel show the median $f_{\star }(t_{\rm bar})$ and median $\mathcal {S}_{\rm bar}$ for each sub-sample, while the black errorbars show the IQR. The horizontal dashed grey line indicates where galaxies are centrally stellar-dominated (above), or DM-dominated (below). Distributions of $\mathcal {S}_{\rm bar}$ for $f_{\star }(t_{\rm bar})$ sub-sample are shown on the upper *x*-axis.

We quantify the local tidal field during bar formation using $\mathcal {S}_{\rm bar}$, defined as the maximum $\mathcal {S}$ within one $t_{\rm dyn}$ before $t_{\rm bar}$; larger values correspond to stronger tidal forces just before bar formation. The main panel of Fig. 5 plots the relationship between $\mathcal {S}_{\rm bar}$ and $f_{\star }(t_{\rm bar})$. The light-coloured squares show that the median value of $\mathcal {S}_{\rm bar}$ increases as the median value of $f_{\star }(t_{\rm bar})$ declines (with a Spearman rank correlation coefficient of $r_{\rm p} = -0.49$). This suggests that while stellar-dominated discs can form bars secularly under relatively weak tidal perturbations (low $\mathcal {S}_{\rm bar}$), increasingly DM-dominated discs require stronger perturbations, and often experience large tidal interactions around the time their bars first form (high $\mathcal {S}_{\rm bar}$).

This is further corroborated by comparing the distributions of $\mathcal {S}_{\rm bar}$ from the most highly stellar- and DM-dominated populations on the top axis (blue and red PDFs). These distributions highlight the diversity of tidal forces bar-forming galaxies experience. The continuous, unimodal, but overlapping distributions of $\mathcal {S}_{\rm bar}$ for different $f_{\rm bar}(t_{\rm bar})$ bins suggest that secular and tidal bars are not always separable, stressing that any threshold between the two is meaningful only in controlled experiments.^4^ Ultimately, the most stellar- and DM-dominated distributions are found to be statistically distinct, further suggesting that bars with different $f_{\rm bar}(t_{\rm bar})$ are not forming under the same processes. Thus, having a stellar-dominant disc is not a prerequisite for bar formation generally, but *is* required for secular bar formation; DM-dominated discs tend to form bars through tides.

Note the significant scatter along the relation, which suggests that some stellar-dominated discs form bars after experiencing large tidal forces. This is expected, as a high $f_{\star }(t_{\rm bar})$ does not preclude a galaxy from experiencing a strong interaction or merger. Furthermore, other factors like the disc properties (e.g. S. Ghosh et al. 2023) or the orbital configuration of the tidal interaction (e.g. E. L. Łokas 2018; N. Peschken & E. L. Łokas 2019) can influence bar formation, increasing the scatter in this plane. The MW provides a possible example: its bar is thought to have formed after a merger, and may have been long-lived (e.g. J. Grady, V. Belokurov & N. W. Evans 2020; A. Merrow et al. 2024; J. L. Sanders et al. 2024). This scenario is consistent with the range of behaviours observed in the stellar-dominated discs of our sample. Similarly, the scatter in Fig. 5 also shows that four marginally DM-dominated discs form bars even in moderately secular conditions when $\mathcal {S}_{\rm bar} \le 0.1$. This emphasises that not all bars can be clearly attributed to one driver; in particular, systems that are only slightly DM-dominated (and thus borderline secularly stable) require only a small tide to tip them into bar formation.

**Figure 6.**
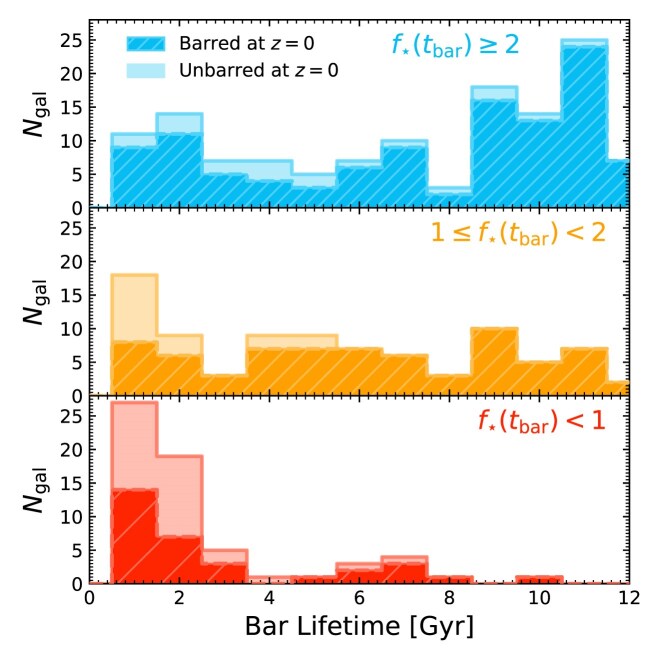
The distribution of bar lifetimes for galaxies in each $f_{\star }(t_{\rm bar})$ bin, with $f_{\star }(t_{\rm bar})$ decreasing from top to bottom. Hatched histograms with dashed outlines show the distribution for galaxies that are still barred at $z=0$, which should be treated as a lower limit. The open histograms with solid outlines show the distribution of galaxies that are unbarred at $z=0$; these are added on top of the hatched histograms.

In Fig. 6, we show the distribution of bar lifetimes for galaxies with different $f_{\star }(t_{\rm bar})$. Hatched distributions represent galaxies that remain barred at $z=0$, for which the lifetimes are lower limits. Open distributions correspond to systems in which the bar no longer exists at $z=0$; in these cases, the bar lifetime is taken to be the time interval during which the galaxy was barred. Bars in stellar-dominated discs are typically long-lived (median $\approx 9,{\rm Gyr}$) and persist to $z=0$, whereas bars in DM-dominated discs are usually short-lived (median $\approx 2,{\rm Gyr}$) and disappear by $z=0$. Indeed, the majority of bars that form with $\mathcal {S}_{\rm bar} \ge 0.2$ last for less than $\lesssim 2\, {\rm Gyr}$ (most of these are DM-dominated, and they all experienced a merger within $\pm 2\, t_{\rm dyn}$ of $t_{\rm bar}$, see Appendix C). Note, however, that bars with high $\mathcal {S}_{\rm bar}$ can be long-lived features if they form in galaxies had high $f_{\star }(t_{\rm bar})$; this suggests that the bar’s lifetime is set more by the disc’s internal properties than the bar formation mechanism. This agrees with previous results showing that bars that formed in isolation in idealized disc galaxy simulations tend to be long-lived (e.g. E. Athanassoula 2013), whereas bars formed tidally or in cosmological settings can exhibit a broader range of bar lifetimes (E. Romano-Díaz et al. 2008; C. Scannapieco & E. Athanassoula 2012; D. G. Algorry et al. 2017; N. Peschken & E. L. Łokas 2019; S. Ansar et al. 2025; F. Fragkoudi et al. 2025).

Why do some bars disappear by $z=0$? We find that roughly half of all bars that are terminated do so after a period of high $\mathcal {S}$, frequently associated with a merger, while the rest display a gradual, secular dampening (with some becoming bulge-dominated systems, see I. D. Gargiulo et al. 2022). Note however that not all mergers lead to the loss of a bar (e.g. I. Martinez-Valpuesta et al. 2017; E. L. Łokas 2018; T. Zana et al. 2018a). Understanding the resilience of bars to some mergers and interactions, but not others, is beyond the scope of this work.

## 5 THE PROPERTIES OF SECULAR AND TIDAL BARS

As shown so far, in our TNG50 sample, bars form in both stellar- and DM-dominated discs, mainly through secular evolution and tidal interactions, respectively. We now examine whether other disc properties influence bar formation, whether they produce bars with different characteristics, and whether bars that formed while stellar- or DM-dominated (and thus predominantly tidally or secularly) can be distinguished in low-*z* galaxies using observables.

### 5.1 The properties of tidal and secular bars

**Figure 7.**
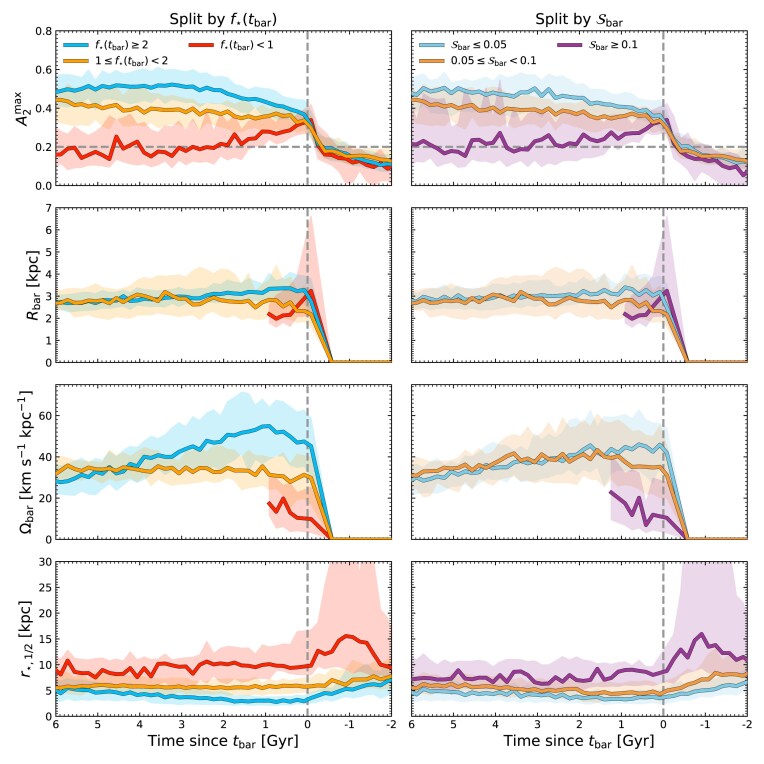
From top to bottom, we show the bar strength, length, pattern speed, and stellar half mass size, respectively, as a function of lookback time normalized by the bar formation time, $t_{\rm bar}$. In the left panel, galaxies that form bars under different $f_{\star }(t_{\rm bar})$ are coloured as in Fig. 2. In the right panel, galaxies are split into roughly equal bins of $\mathcal {S}_{\rm bar}$, with $\mathcal {S}_{\rm bar} \le 0.05$ in light blue, $0.05 < \mathcal {S}_{\rm bar} \le 0.1$ in light orange, and $\mathcal {S}_{\rm bar} \ge 0.1$ in purple. Shaded regions indicate the IQR of each population. In the upper panels, we show as a dashed grey line the $A_{2}^{\rm max} = 0.2$ threshold used to identify bars.

In Fig. 7, from top to bottom we show the evolution of the bar strength ($A_2^{\rm max}$), bar length ($R_{\rm bar}$), and bar pattern speed ($\Omega _{\rm bar}$), and the stellar half-mass radius ($r_{\star , 1/2}$). In the left column, the median trends for galaxies with different $f_{\star }(t_{\rm bar})$ are coloured as before, with the blue, orange, and red lines indicating galaxies that formed their bars when they were stellar-dominated, marginally stellar-dominated, and DM-dominated, respectively. In the right column, we split the sample into three bins of $\mathcal {S}_{\rm bar}$ with roughly equal numbers of galaxies, which approximately follow the median $\mathcal {S}_{\rm bar}$ of the populations selected on $f_{\star }(t_{\rm bar})$: $\mathcal {S}_{\rm bar} \le 0.05$ in light blue, $0.05 < \mathcal {S}_{\rm bar} \le 0.1$ in light orange, and $\mathcal {S}_{\rm bar} \ge 0.1$ in purple. The samples split by $\mathcal {S}_{\rm bar}$ exhibit similar evolutionary tracks as those split by $f_{\star }(t_{\rm bar})$. To facilitate comparisons between bars at similar evolutionary stages, we define $t=0$ as the time of bar formation. In the middle two rows, galaxies contribute to the median only while they are barred, and curves are shown only when at least 20 barred systems are available. By contrast, $A_{2}^{\rm max}$ and $r_{\star , 1/2}$ are shown for all galaxies, regardless of whether they are barred or not. In Appendix D, we show the unnormalized bar properties as a function of $M_{\star }$ at $t_{\rm bar}$ and at $z=0$.

The top row of Fig. 7 shows that the bar strength evolves differently for galaxies with different $f_{\star }(t_{\rm bar})$ or $\mathcal {S}_{\rm bar}$. At $t_{\rm bar}$, all populations show comparable $A_{2}^{\rm max}$ by construction, but diverge thereafter. Stellar-dominated discs, or those that experienced weak tidal perturbations near $t_{\rm bar}$, tend to form bars that grow stronger over a short time interval. In contrast, bars in DM-dominated discs, or those that form coincident with a large tidal perturbation, show a gradual decline in $A_{2}^{\rm max}$ after $t_{\rm bar}$, as expected given the short lifetimes found for these bars in Fig. 6.

**Figure 8.**
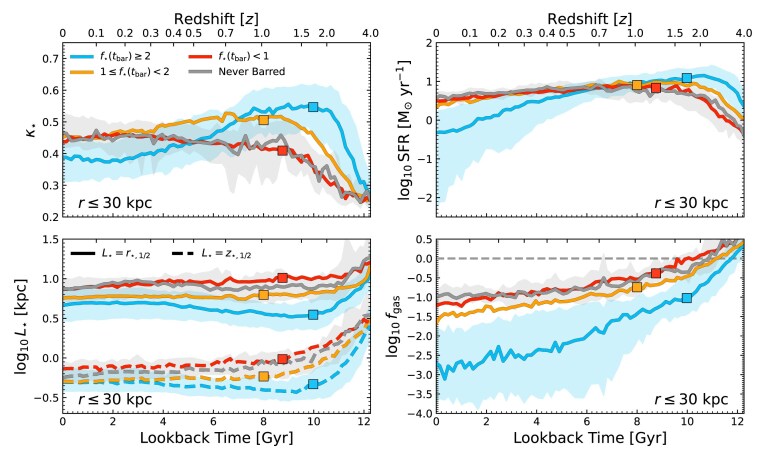
We show the time evolution of various galaxy morphology indicators for galaxies that form bars under different $f_{\star }(t_{\rm bar})$, coloured as in Fig. 2. We show the fraction of energy invested in corotation, $\kappa _{\star }$ (within $r\le 30\, {\rm kpc}$; upper left), star formation rate (measured over the past $50\, {\rm Myr}$ and within $r\le 30\, {\rm kpc}$; upper right), the stellar half mass height and radius (measured from the absolute value of stellar heights above the mid-plane, and on particles within $r\le 30\, {\rm kpc}$, respectively; lower left), and the gas-to-stellar mass ratio, $f_{\rm gas}$ (measured within $r\le 30\, {\rm kpc}$; lower right). Coloured squares indicate the median bar formation time for these populations. Shaded areas show the IQR of select sub-samples.

The panel second from the top in Fig. 7 reveals additional differences in the evolution of the bar length. S. Ghosh & P. Di Matteo (2024) showed that our choice to consider both the Fourier amplitude and phase in our measurements of $R_{\rm bar}$ minimizes the bias introduced by tidally triggered spiral arms, though $R_{\rm bar}$ should still be interpreted cautiously. During the formation of bars in stellar-dominated discs, or in discs with low $\mathcal {S}_{\rm bar}$, the bar length reaches its peak directly after $t_{\rm bar}$, and then remains roughly constant for the rest of its lifetime. In contrast, during bar formation in DM-dominated discs, or in discs with high $\mathcal {S}_{\rm bar}$ tidal interactions, the bar exhibits a rapid increase in length (at $t_{\rm bar}$, many of the longest bars in our sample are found in these galaxies), followed by a gradual decline as many of these bars disappear. Thus, many of the longest observed bars, if viewed near the time of bar formation, may be those formed in DM-dominated galaxies, and often formed as a result of significant tidal interactions.

The panel second from the bottom in Fig. 7 shows that the evolution of the bar pattern speed is related to $f_{\star }(t_{\rm bar})$: bars in more stellar-dominant discs reach higher peaks in $\Omega _{\rm bar}$. Following this peak, these bars exhibit a declining pattern speed, consistent with both theoretical expectations and previous simulations (e.g. S. Tremaine & M. D. Weinberg 1984; M. D. Weinberg 1985; J. Zavala, T. Okamoto & C. S. Frenk 2008). On the other hand, bars that form in progressively DM-dominated discs exhibit lower peaks in pattern speed. The most DM-dominated discs, for instance, show the lowest $\Omega _{\rm bar}$ before their bars are terminated. The left column shows that these trends are somewhat $\mathcal {S}_{\rm bar}$ agnostic: galaxies with $S_{\rm bar} \le 0.1$ (i.e. light blue and light orange lines), all show a similar pattern speed evolution, and are distinguished more when split by $f_{\star }(t_{\rm bar})$. However, galaxies with high $\mathcal {S}_{\rm bar} > 0.1$ still show low $\Omega _{\rm bar}$, consistent with their large lengths (E. Athanassoula 2003). These results broadly echo those of Y. Zheng & J. Shen (2025), who found that tidally induced bars that were otherwise stable to secular bar formation rotated more slowly than those that were secularly unstable (see also I. Martinez-Valpuesta et al. 2017). This also agrees predictions of T. Miwa & M. Noguchi (1998), who suggest that all tidal bars should show lower $\Omega _{\rm bar}$ than secular bars (e.g. E. L. Łokas et al. 2014; E. L. Łokas 2018; A. R. Pettitt & J. W. Wadsley 2018). However, it seems that differences in $\Omega _{\rm bar}$ are driven more by the differences in the internal properties of galaxies, particularly $f_{\star }$, than by the different formation mechanisms, though a strongly tidally perturbed bar may be identifiable by its long $R_{\rm bar}$ and slow $\Omega _{\rm bar}$ for short periods of time after $t_{\rm bar}$.

It is important to view these results in the context of the galaxy size: the bottom panel of Fig. 7 shows that as $f_{\star }(t_{\rm bar})$ increases and $\mathcal {S}_{\rm bar}$ decreases, galaxies become more compact. As a consequence, bars formed after strong tidal interactions, or in DM-dominated galaxies, are shorter relative to the disc size than those formed otherwise. For instance, when measured at $t_{\rm bar}$, $R_{\rm bar}/r_{\star ,1/2} = 1.30^{+0.33}_{-0.46}$ in the secularly forming, $f_{\star }(t_{\rm bar}) \ge 2$ sample, but $R_{\rm bar}/r_{\star ,1/2} = 0.31^{+0.16}_{-0.11}$ in the tidally forming, $f_{\star }(t_{\rm bar}) < 1$ sample (the upper and lower indices report the $\pm 1\sigma$ error); thus the relative sizes are statistically distinct. Similarly, given their extended sizes, DM-dominated galaxies, or those formed after high $\mathcal {S}_{\rm bar}$, produce bars that rotate faster relative to the disc rotational velocity than stellar-dominated, low $\mathcal {S}_{\rm bar}$ galaxies. Note however, that if bars can persist for $\approx 4\, {\rm Gyr}$, the differences between these samples are reduced, and all surviving bars tend towards $R_{\rm bar}/r_{\star ,1/2}\approx 0.5$, and $\Omega _{\rm bar}/\Omega _{\rm disc} \approx 1$ (where $\Omega _{\rm disc}$ is the disc rotational velocity at $r_{\star ,1/2}$). This suggests that, once embedded and stable, bars grow coevally with their discs (e.g. T. Kim et al. 2021) regardless of the formation mechanism.

Ultimately, while bars formed in stellar-dominated discs and DM-dominated discs (or formed after weak tidal perturbations versus those formed after strong tidal perturbations) show distinct trends in strength, length, and pattern speed, these differences become increasingly subtle with time, and are further complicated by differences in galaxy properties. Once a bar is well-established, its properties alone are likely insufficient to unambiguously determine its formation mechanism. Thus, early evolution can hint at a bar’s origin, especially when paired with contextual information about the host galaxy or the nearby environment, but the bars themselves do not always retain a clear imprint of their formation history.

### 5.2 Galaxy properties

While our analysis confirms that strongly self-gravitating disc galaxies are more susceptible to secular bar formation (see also G. Efstathiou et al. 1982; J. Bland-Hawthorn et al. 2023; M. Frosst et al. 2024), other properties like disc thickness (e.g. A. Klypin et al. 2009; M. Aumer & J. Binney 2017; S. Ghosh et al. 2023) or gas fraction (e.g. I. Berentzen et al. 2004; F. Bournaud et al. 2005) may also be important.

To investigate the link between galaxy structure and bar formation, in Fig. 8 we present the redshift evolution of four key quantities that trace a galaxy’s structure and susceptibility to bar formation in discs with different $f_{\star }(t_{\rm bar})$ (but note that very similar trends are identified if plotted with the $\mathcal {S}_{\rm bar}$ selection presented in Fig. 7). A striking feature is the link between $f_{\star }(t_{\rm bar})$ and the assembly of galaxy stellar structure. While it is well understood that barred galaxies assemble their mass faster than unbarred galaxies (found in tng, e.g. Y. Rosas-Guevara et al. 2022; S. Khoperskov et al. 2024, but also recent observations, e.g. A. Fraser-McKelvie et al. 2020), we see now that galaxies that are more stellar-dominated at $t_{\rm bar}$ assemble their discs earlier than galaxies that are more DM-dominated at $t_{\rm bar}$. This is indicated by higher $\kappa _{\star }$ at high *z*, but also by how these galaxies become thinner and more compact at earlier times. In contrast, the galaxies that form bars in DM-dominated discs tend to assemble their discs later, and, even by $z=0$, remain less compact than galaxies that formed bars with higher $f_{\star }(t_{\rm bar})$. These structural trends are consistent with the idea that bars in stellar-dominated discs, often formed after low $\mathcal {S}_{\rm bar}$, form predominantly in thin, kinematically cold discs prone to internal instabilities, whereas bars in DM-dominated discs form in more stable discs that require external tidal perturbations and high $\mathcal {S}_{\rm bar}$ to induce bar formation (e.g. A. Klypin et al. 2009; M. Aumer & J. Binney 2017; F. Fragkoudi et al. 2017; S. Ghosh et al. 2023).

Interestingly, while the most highly stellar-dominated galaxies assemble their discs earliest, and thus form their bars earliest, by $z=0$ they have the lowest $\kappa _{\star }$ of all sub-samples. We speculate that this is related to the presence of the long-lived bars found in these galaxies, which should increase the central velocity dispersion as they evolve (e.g. M. Das et al. 2008). T. Zana et al. (2022) also suggest that the presence of bars may lower $\kappa _{\star }$. It is possible that some bars in MW-mass disc galaxies could be missed due to $z=0$ selections on this parameter, however, in our case, we note that very few ($< 10$) galaxies that are barred are lost due to our selection on $\kappa _{\star }$ at $z=0$.

Another notable (but related) distinction emerges in the SFR and gas content of these galaxies. Galaxies that go on to form bars in highly stellar-dominated discs are significantly more gas-poor than their DM-dominated counterparts, even at early times.^5^ They also tend to be more highly star forming at high redshifts, but more quenched by $z=0$ – as a consequence, they are often more stellar *in situ* dominated, having assembled most of their mass through star formation. As $f_{\star }(t_{\rm bar})$ gets lower, the galaxies retain higher $f_{\rm gas}$ values over time, and exhibit flatter star formation histories. Given the connections we have presented between $f_{\star }(t_{\rm bar})$ and $\mathcal {S}_{\rm bar}$, this may be consistent with previous work showing that gas-rich discs are less prone to secular bar formation (e.g. E. Athanassoula, R. E. G. Machado & S. A. Rodionov 2013), although recent work has suggested that $f_{\rm gas}$ makes little difference at fixed $f_{\star }$ (J. Bland-Hawthorn et al. 2024; F. Fragkoudi et al. 2025). It has also been shown that bars in gas-rich galaxies tend to be more shortly lived (e.g. I. Berentzen et al. 2004; F. Bournaud et al. 2005; J. Bland-Hawthorn et al. 2024), in agreement with our results in Fig. 6. It is possible that the combination of high gas fractions and external perturbations in DM-dominated bar hosts may weaken or ultimately destroy nascent bars through central mass concentration and induced turbulence (e.g. D. Friedli & W. Benz 1993; F. Bournaud et al. 2005; N. Peschken & E. L. Łokas 2019). Ultimately, caution is warranted when interpreting trends with $f_{\rm gas}$, as they may be influenced by strong super massive black hole (SMBH) feedback in the tng model, often associated with galaxies that form secular bars in this mass range, potentially complicating direct causal interpretations (M. Frosst et al. 2025).

Finally, galaxies that never form bars are on average the most extended, thickest, most centrally DM-dominated, highly star forming, gas-rich galaxies in our entire sample at all times (see also F. Fragkoudi et al. 2020; S. Lu, M. Du & V. P. Debattista 2025). These galaxies tend to form their discs later than the galaxies that form bars, though by $z=0$, they tend to have the largest fraction of stellar mass in ‘disc-like’ orbits, with high $\kappa _{\star }$. Interestingly, however, the properties of unbarred galaxies are usually indistinguishable from the properties of DM-dominated bar-forming galaxies. Their resistance to bar formation is likely due to a combination of factors: strong central DM-dominance stabilises the disc against tidal perturbations (see Fig. 2), and it is possible that these galaxies simply never experience an interaction with the correct properties to trigger bar formation in their otherwise stable discs.

**Figure 9.**
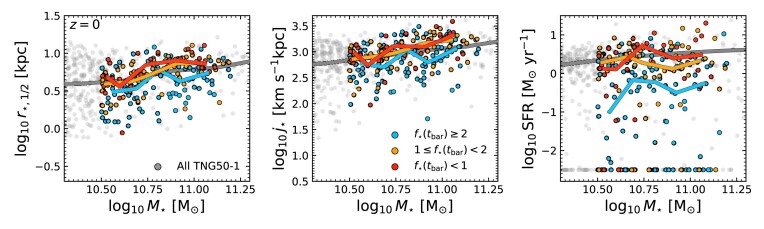
We show three scaling relations for our sample at $z=0$. From left to right, we show the size–mass relation, Fall relation, and star formation main sequence (SFMS). Galaxies are coloured based on $f_{\star }(t_{\rm bar})$, as in Fig. 2, but are only coloured if they are currently barred at these redshifts. For comparison, all TNG50 galaxies are included as light grey points. The medians of each population are shown as thick lines of the same colours.

A possible criticism of Fig. 8 is that we include all bars regardless of bar lifetimes, as not all galaxies in each bin remain barred until $z=0$ (e.g. Fig. 6). To address this, in Fig. 9 we show (from left to right) the size ($r_{\star ,1/2}$)–mass relation, the specific angular momentum ($j_{\star }$)–mass relation, and the SFMS at $z=0$; $j_{\star }$ and star formation rate (SFR) measured within $r\le 30\, {\rm kpc}$, with SFR measured over the past $50\, {\rm Myr}$. Now, the galaxies in the $f_{\star }(t_{\rm bar})$ sub-samples are included only if they are actively barred at $z=0$. The median trends for the actively barred galaxies are shown as lines of the same colours. For comparison, all TNG50 galaxies are included as grey points, with the light grey line indicating the median.

As expected from Fig. 8, by $z=0$ actively barred galaxies in the most stellar-dominated discs at $t_{\rm bar}$ have lower $r_{\star ,1/2}$, $j_{\star }$, and SFR at fixed mass, even when compared to the entire TNG50 median. However, the differences between the moderately stellar-dominated, DM-dominated, and unbarred populations are less noticeable. Ultimately, while clear separations between the $f_{\star }(t_{\rm bar})$ sub-samples are not possible in these spaces, nearly all actively barred galaxies at $z=0$ with compact $r_{\star ,1/2}$, low $j_{\star }$, and significant SFR quenching have $f_{\star }(t_{\rm bar}) \ge 2$. If observed, bars in such low$-z$ galaxies could be tentatively classified as secular. This once again makes it clear that distinguishing between bar formation mechanisms at any given redshift is very difficult, especially without access to individual bar histories and formation times.

Fianlly, we emphasize that our results are moderated by a number of caveats. Notably, bar formation in TNG50 is correlated with the onset of strong SMBH feedback in this mass range (M. Frosst et al. 2025), which may bias the connections between disc and bar properties, particularly bar lifetimes and $f_{\star }$. Moreover, our analysis focused on a single simulation and a carefully selected sample of galaxies that are discs at $z=0$. This introduces potential biases, particularly as other large-volume simulations with coarser resolution have reported a greater prevalence of tidally induced bars (e.g. N. Peschken & E. L. Łokas 2019) or drastically lower bar fractions generally (e.g. J. Reddish et al. 2022). In addition, we have studied only one specific feedback model; prior work shows that the nature and frequency of bars can vary with the feedback prescriptions (T. Zana et al. 2019). Our results therefore assume that TNG50 galaxies have sufficient resolution at all times to model both secular and tidally induced bar formation, and that the sub-grid physics models are realistic.

## 6 SUMMARY AND CONCLUSIONS

In this work, we studied the evolution of 307 disc galaxies from TNG50 with roughly MW-mass at $z=0$ as a function of their central stellar-to-DM mass fraction ($f_{\star }$) at the time of bar formation ($t_{\rm bar}$). Preliminary visual inspection suggested that some of these bars were formed during tidal interactions, rather than secular processes, particularly if their discs were centrally DM-dominated at $t_{\rm bar}$. To quantify this, we introduced a new metric for measuring the tidal fields acting on galaxies, $\mathcal {S}$ (equation 7), which considers not only the tidal forces imparted simultaneously by nearby passing and merging galaxies, but the entire environment surrounding the galaxy. We used this metric to measure the tidal field present prior to bar formation, $\mathcal {S}_{\rm bar}$. Below, we highlight our main results, focusing on galaxies with only one bar episode (nearly 66 per cent of the total sample, see Fig. 1).

- Many bars form in centrally stellar-dominated discs [$f_{\star }(t_{\rm bar}) \ge 1$], but $\approx 24$ per cent of our sample form bars while DM-dominated (right panel, Fig. 2), and thus while their discs are secularly stable against internally driven bar formation. Furthermore, the galaxies central mass assembly is highly segregated by $f_{\star }(t_{\rm bar})$: as $f_{\star }(t_{\rm bar})$ decreases, bars form later, $f_{\star }$ peaks lower, and the growth rate of $f_{\star }$ decreases (left panel, Fig. 2). Interestingly, the galaxies that form bars in the most DM-dominated discs have similar $f_{\star }$ at all times to galaxies that never form bars.
- We found a moderate negative correlation between $f_{\star }(t_{\rm bar})$, and $\mathcal {S}_{\rm bar}$, the tidal force a galaxy experiences prior to bar formation. Generally, as galaxies become more centrally DM-dominated and secularly stable against bar formation, they require increasingly large tidal forces to initiate a bar episode (Fig. 5). As a result, stellar-dominated discs form bars primarily through secular evolution, while DM-dominated discs more frequently form bars after strong tidal interactions, during mergers or flybys. However, exceptions to this rule are possible, with stellar-dominated, secularly unstable discs occasionally forming bars after strong tidal interactions, and DM-dominated, secularly stable discs occasionally forming bars after weak interactions – this makes distinguishing bar origins particularly challenging.
- Furthermore, bars form across a broad, unimodal, continuous distribution of tidal environments (top distributions, Fig. 5). This indicates that secular and tidal bar formation cannot be arbitrarily separated in a cosmological context. Additionally, bars never form in perfectly isolated conditions (i.e. we never measure $\mathcal {S_{\rm bar}} = 0$) as often assumed in idealized simulations of secular bar formation.
- Centrally stellar-dominant bars are frequently long-lived, while DM-dominant bars are often transient features (Fig. 6). However, if a stellar-dominated galaxy forms a bar due to a tidal interaction, it also tends to be long-lived. This indicates that the disc’s internal properties are more important for determining bar lifetimes than the bar formation mechanism.
- The properties of bars themselves are not also reliable indicators of their formation pathway or $f_{\star }$ history, unless measured within a few Gyr of $t_{\rm bar}$ (Fig. 7). First, differences in bar properties seem to be driven more by the internal disc properties, rather than the formation mechanism. Second, while secular/stellar-dominated and tidal/DM-dominated bars might initially show distinct trends in strength, length, and pattern speed, these differences become increasingly subtle with time. For example, if caught near the time of bar formation, bars in both DM-dominated discs, or discs with large $\mathcal {S}_{\rm bar}$, are among the longest, most slowly rotating bars in our sample. Once a bar is well-established, its properties alone are insufficient to unambiguously determine its formation mechanism.
- The evolution of discs that form different bars is distinct (Fig. 8): galaxies that form bars in stellar-dominated discs exhibit compact, thin, quenched, gas-poor discs which form their discs relatively early ($z \gtrsim 2$). Conversely, galaxies that form bars in DM-dominated discs – or never form bars – tend to be thicker, more extended, more gas-rich and highly star forming, and only become disc-like at later times ($z \lesssim 1$). That the disc properties of DM-dominated bars and galaxies that never form bars are similar highlights the importance of the tidal interactions for bar formation in DM-dominated discs, and the importance of the internal disc properties for bar formation in stellar-dominated discs.
- Despite the differences in the evolutionary histories, at any fixed redshift it is challenging to distinguish between discs that formed bars through different mechanisms, or while under different $f_{\star }(t_{\rm bar})$, as the individual evolutionary histories significantly complicate the picture (Fig. 9). However, at $z=0$ the galaxies that formed bars in the most stellar-dominated discs do have smaller sizes, lower specific angular momentum, and lower star formation rates, on average.

Looking ahead, future studies should test the robustness of these findings across cosmological simulations with varying resolutions and feedback models. Higher output cadence and multisimulation comparisons will be valuable for disentangling the causality between strength of tidal events, the onset of bar formation, and the assembly of disc structure. Observationally, upcoming integral field unit (IFU) surveys of disc galaxies (particularly those capable of resolving stellar kinematics, e.g. timer; D. A. Gadotti et al. 2019, PHANGS-MUSE; E. Emsellem et al. 2022, and geckos; J. de Sande et al. 2023; A. Fraser-McKelvie et al. 2025, among others) may be able to constrain bar origins through stellar population gradients (see A. Molaeinezhad et al. 2017), bar age estimates (e.g. C. Sá-Freitas et al. 2023, 2025), or other structural diagnostics. Ultimately, understanding how, when, and why bars form will provide crucial insight into the role they play in galaxy formation and evolution in a cosmological context.

## Figures and Tables

**Figure A1.**
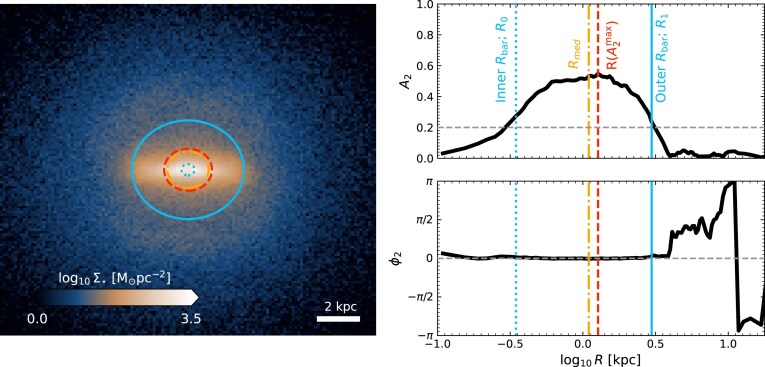
The face-on stellar surface mass density (left; as in Fig. 3), and radial $A_{2}(R)$ and $\phi _{2}(R)$ profiles, (right, top and bottom, respectively). Light blue dotted and solid lines indicate the inner and outer bar radii, $R_{0}$ and $R_{1} = R_{\rm bar}$, respectively. The orange dash–dotted line shows $R_{\rm med}$, the median radius between $R_{0}$ and $R_{1}$. The dashed red line shows the radius of $A_{2}^{\rm max}$.

**Figure C1.**
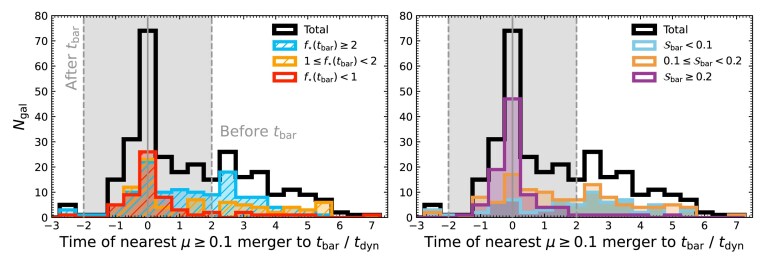
Histogram of the time relative to $t_{\rm bar}$, normalized by $t_{\rm dyn}$, of the nearest merger with stellar mass ratio $\mu \ge 0.1$. The total distribution is shown as a thick black line. Histograms for bars formed in different $f_{\star }(t_{\rm bar})$ and different $\mathcal {S}_{\rm bar}$ are shown in the left and right panels, respectively.

**Figure C2.**
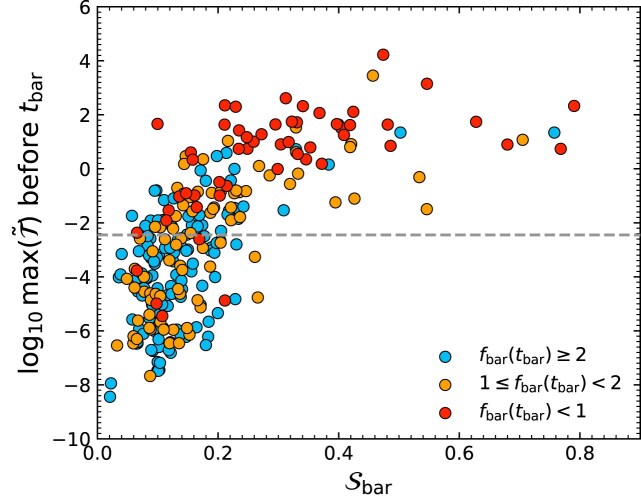
Scatter between $\max (\tilde{\mathcal {T}})$ and $\mathcal {S}_{\rm bar}$ for galaxies in our sample that form one bar episode. Points are coloured according to $f_{\star }(t_{\rm bar})$, as before. The horizontal grey dashed line denotes a threshold previously used to delineate tidal and secular bars (P. D. López et al. 2024; S. Ansar et al. 2025).

**Figure D1.**
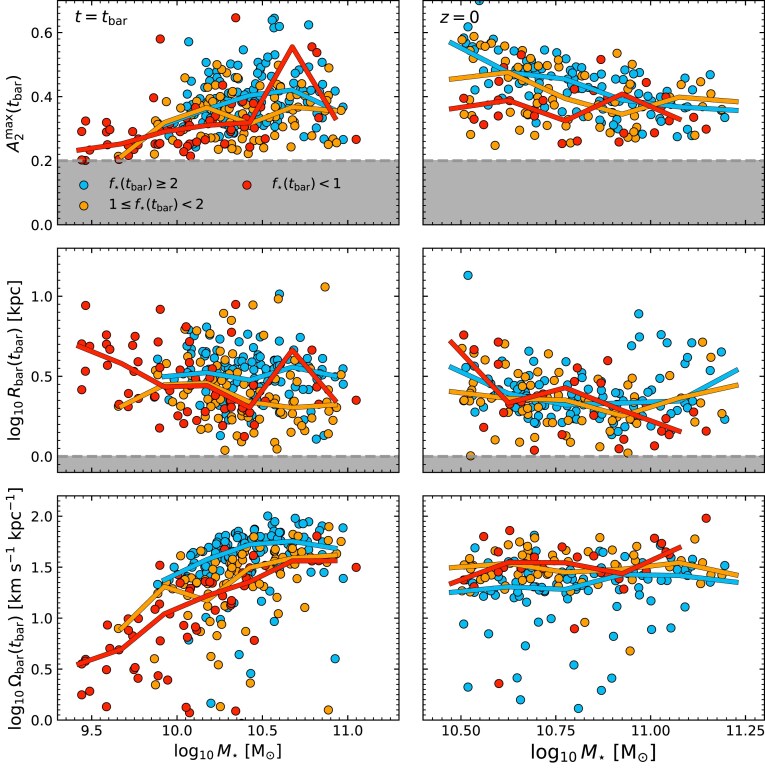
From top to bottom we show the bar strengths, lengths, and pattern speeds as a function of their stellar mass at the time of bar formation, $t_{\rm bar}$. Points are coloured according to whether $f_{\star }(t_{\rm bar})$, as before. Lines of the same colour indicate the median value of these populations as a function of stellar mass. Shades regions indicate the limits we require for a bar to have formed.

## Data Availability

The tng simulation outputs used for our analysis are publicly available at http://www.tng-project.org; see D. Nelson et al. (2019), A. Pillepich et al. (2018a) for further information. The TNG50 MW/M31-like sample is also publicly available at the above link, but A. Pillepich et al. (2024) for details. A simple python script to calculate the total tidal tensor, $\mathbb {T}$, or the tidal field strength, $\mathcal {S}$, can be found at https://github.com/mattfrosst/tidalfields/. Additional data or analysis codes can be made available upon reasonable request.

## Footnotes

1 The value of $4\, r_{\star ,1/2}$ is suitable for our purposes because it encompasses the stellar disc. Our tests reveal that varying $r_{\rm t}$ by a factor 2 does not significantly change the results. However, $r_{\rm t} \lesssim 2r_{\star ,1/2}$ causes the disc itself to dominate the tidal field in many cases, and $r_{\rm t}\gtrsim 8r_{\star ,1/2}$ would exclude many tidal effects of nearby and merging satellites. There is no optimal choice of $r_{\rm t}$ for all galaxies at all times, in all environments, but $r_{\rm t} = 4r_{\star ,1/2}$ is a consistent value for our analysis.

2 Because $\mathcal {T} \propto r^{-3}$, the choice of outer radius does not affect our results, provided it exceeds a few times $r_{\rm t}$.

3 A python script to measure $\mathcal {T}$ and $\mathcal {S}$ from simulation data are available at https://github.com/mattfrosst/tidalfields/.

4 Others have attempted to justify other, comparable methods to define tidal and secular bars in cosmological simulations. We show in Appendix C that arbitrary thresholds in $\mathcal {S}_{\rm bar}$ can be roughly consistent with these methods.

5 Note that, while there is no statistical difference in $M_{\star }$ between the different $f_{\star }(t_{\rm bar})$ bins at $z=0$, stellar-dominated discs do assemble their stellar mass earlier than DM-dominated discs, which may influence these results.

## APPENDIX A: MEASURING BAR PROPERTIES

Much of our analysis relies on the accurate measurement of bar properties. We have established that our measurements use a Fourier decomposition to determine $A_{2}$ (equation 3) and $\phi _{2}$ (equation 4). These properties are measured as a function of radius, calculated on all stellar particles in cylindrical radial bins centred on the galaxies centre of potential (measured from all bound particles, and, in practice, at the centre of the DM halo). We include exactly $N_{\rm part} = 10^4$ disc particles per radial bin to keep the Poisson noise, scaling as $\sigma _{A_{2}} = 1/\sqrt{N_{\rm part}}$, at 1 per cent while maintaining good radial resolution at our stellar mass range in TNG50 – higher $N_{\rm part}$ reduces noise but lowers the spatial resolution leading to less reliable bar property measurements. The most distant radial bin may contain less than $N_{\rm part}$, but in practice this bin is never considered in our analysis. Following W. Dehnen et al. (2023), after determining the initial number of bins, $N_{\rm bin}$, we add an additional $N_{\rm bin}-1$ intermediate bins between the medians of all initial bins, leaving us with $2N_{\rm bin}-1$ overlapping bins in total. Within each bin, we calculate the $A_{2}(R)$ and $\phi _{2}(R)$ used in this work.

In Fig. A1, we show the face-on surface mass density projection, $\Sigma _{\star }$, of a barred galaxy, as well as the radial $A_{2}(R)$ and $\phi _{2}(R)$ profiles in the top and bottom rows of the left panel. The bar length, $R_{\rm bar}$, is shown as a solid blue line/circle, and is identified as the region around $A_{2}^{\rm max}$ (dashed red line/circle) within which $\phi _{2}(R)$ deviates from $\phi _{2}^{\rm max}$ by $\le \pm 10^{\circ }$ while $A_{2}(R) \ge A_{2}^{\rm max}/2$, following the procedure outlined in W. Dehnen et al. (2023). This region therefore has both an inner and outer radius, labelled $R_{0}$ and $R_{1}$ and shown as dotted and solid vertical blue lines/circles in Fig. A1; when quoting a value for the bar radius we simply quote the *outer* radius, i.e. $R_{1} = R_{\rm bar}$. We require $R_{\rm bar} > 1\, {\rm kpc}$, otherwise we do not identify a bar regardless of the value of $A_{2}^{\rm max}$. The face-on projection of the example galaxy shows that this method produces an $R_{\rm bar}$ which precisely captures the bars extent. Other methods can find similar bar lengths (e.g. S. Ghosh & P. Di Matteo 2024), but we caution against assuming $R_{\rm bar} = R(A_{2}^{\rm max})$, which, as seen in Fig. A1, produces a severe underestimate.

Measuring the bar pattern speed is slightly more complicated. First, note that $\phi _{2}$ accurately determines a bar’s orientation (evidenced by Fig. A1, where we have aligned the bar with the *x*-axis). Second, the bar pattern speed, $\Omega _{\rm bar}$, can be determined instantaneously from the time derivative of $\phi _{2}$,

(A1)
\begin{eqnarray*}
\Omega _{\rm bar} = \frac{\mathrm{ d} \phi _{2}}{\mathrm{ d} t} = \sum _{j} \frac{\partial \phi _{2}}{\partial x_{j}} \frac{\mathrm{ d} x_{j}}{\mathrm{ d}t},
\end{eqnarray*}

where $x_{j}$ are the particle positions. However, W. Dehnen et al. (2023) noted critically that the net particle flux on the radial bins must be considered when calculating this derivative, otherwise $\Omega _{\rm bar}$ will have systematic errors of $5{-}20$ per cent. This bias can be minimized by weighing stellar particles with a window function, such as

(A2)
\begin{eqnarray*}
W(R) = (1-Q)^2(1 + 2Q), {\rm where \, } Q = \frac{R^2-R_{\rm m}^2}{R_{\rm e}^2-R_{\rm m}^2},
\end{eqnarray*}

and $R_{\rm med}$ is the middle of the bar region, while $R_{\rm e} = R_{0}$ if $R < R_{\rm med}$ and $R_{\rm e} = R_{1}$ otherwise. Applying this window function to the initial Fourier decomposition, we get

(A3)
\begin{eqnarray*}
\mathcal {A_{\rm m}} \equiv \frac{\sum _j W(x_{j}) M_{j} e^{mi\theta _{j}}}{\sum _j M_{j}},
\end{eqnarray*}

such that the bar strength is,

(A4)
\begin{eqnarray*}
|A_{\rm m}|= \frac{\sqrt{B_{m}^2 + C_{m}^2}}{\sum _{j} M_{j}}.
\end{eqnarray*}

Here,

(A5)
\begin{eqnarray*}
B_{m} = \sum _{j} M_{j} W(x_{j}) \sin (m \theta _{j}), \mathrm{and,}
\end{eqnarray*}

(A6)
\begin{eqnarray*}
C_{m} &= \sum _{j} M_{j} W(x_{j}) \cos (m \theta _{j}).
\end{eqnarray*}

Therefore, $\phi _{2}$ and $\Omega _{\rm bar}$ can be expressed by setting $m=2$ as

(A7)
\begin{eqnarray*}
\phi _{2} = \arg (\mathcal {A}_{\rm 2}) = \frac{1}{2} \arctan \left(\frac{B_{2}}{C_{2}} \right)
\end{eqnarray*}

and

(A8)
\begin{eqnarray*}
\Omega _{\rm bar} = \frac{\mathrm{ d}\phi _{2}}{\mathrm{ d}t} = \frac{C_{2} \dot{C_{2}}-B_{2}\dot{B_{2}}}{2(C_{2}^2 + B_{2}^{2})},
\end{eqnarray*}

where $\dot{B_{2}}$ and $\dot{C_{2}}$ are the time derivatives of $B_{2}$ and $C_{2}$, respectively. This provides a simple way to measure the instantaneous bar pattern speed for any galaxy at any snapshot. We point the reader to W. Dehnen et al. (2023) for more details.

## APPENDIX B: DERIVATION OF TIDAL TENSOR

This section shows the derivation of equations (5) and (6). Consider a system of mass $M_t$ subjected to an external point-perturber of mass *m* located at position $\mathbf {r}$, relative to the centre-of-mass of the system. The gravitational acceleration at any point $\mathbf {x}$ due to this perturber is

(B1)
\begin{eqnarray*}
\boldsymbol {A}(\boldsymbol {x}) = \frac{G m}{|\boldsymbol {r}-\boldsymbol {x}|^{3}}(\boldsymbol {r}-\boldsymbol {x}),
\end{eqnarray*}

where *G* is the gravitational constant. By definition, the tidal acceleration $\boldsymbol {A}_{\rm t}(\boldsymbol {x})$ is the $\boldsymbol {x}$-dependent correction term relative to the centre-of-mass acceleration $\boldsymbol {A}(0)$,

(B2)
\begin{eqnarray*}
\boldsymbol {A}(\boldsymbol {x}) = \boldsymbol {A}(0) + \boldsymbol {A}_{\rm t}(\boldsymbol {x}).
\end{eqnarray*}

Let us Taylor-expand $\boldsymbol {A}_{\rm t}(\boldsymbol {x})$ about $\mathbf {x}=0$,

(B3)
\begin{eqnarray*}
\boldsymbol {A}_{\rm t}(\boldsymbol {x}) = \mathbb {J}\, \boldsymbol {x} + \mathcal {O}(x^2),
\end{eqnarray*}

with the Jacobian $\mathbb {J}$ given by

(B4)
\begin{eqnarray*}
J_{ij} = \left. \frac{\partial a_{i}}{\partial x_{j}} \right|_{x=0} = \frac{Gm}{r^3}\left[3\frac{r_i r_j}{r^2}-\delta _{ij} \right],
\end{eqnarray*}

where $\delta _{ij}$ is the Kronecker delta, or, in matrix notation,

(B5)
\begin{eqnarray*}
\mathbb {J} = \frac{Gm}{r^3}\left[3 \frac{\boldsymbol {r} \otimes \boldsymbol {r}}{r^2}-\mathbb {I}\right],
\end{eqnarray*}

where $\otimes$ denotes the outer product and $\mathbb {I}$ is the identity matrix. The matrix $\mathbb {J}$ is a symmetric and trace-free tensor with eigenvalues $Gmr^{-3}(+2, -1, -1)$. The eigenvector associated with the positive eigenvalue points along $\boldsymbol {r}$, while the orthogonal plane is associated with the two degenerate negative eigenvalues. This leads to the common picture of tides, such as lunar tides causing two symmetric ocean bulges pointing towards and away from the moon with a corresponding compression in the orthogonal plane.

In the context of galaxies spanning a range of masses and hence a range of self-gravity, it makes sense to normalize the tidal acceleration by a reference acceleration linked to the galaxy mass. Since the galaxy-system is defined as the collection of all matter contained in a sphere of radius $r_{\rm t}$ centred on the galaxy (see Section 4.1), the most natural reference acceleration is the galaxy’s spherically averaged self-acceleration on this spherical boundary, $A_{\rm s}=GM_{\rm t}r_{\rm t}^{-2}$. We define the dimensionless tidal acceleration, $\boldsymbol {a}_{\rm t}$, as the linear term of $\boldsymbol {A}_{\rm t}$ normalized by $A_{\rm s}$, hence

(B6)
\begin{eqnarray*}
\boldsymbol {a}_{\rm t}(\boldsymbol {x}) = \frac{m r_{\rm t}^2}{M_{\rm t} r^3}\left[3 \frac{\boldsymbol {r} \otimes \boldsymbol {r}}{r^2}-\mathbb {I}\right]\, \boldsymbol {x}.
\end{eqnarray*}

Generalizing this expression to the acceleration caused by *N* point masses $k=1...N$ is straightforward by virtue of the additivity of forces: it suffices to replace *m* for $m_k$ and $\boldsymbol {r}$ for $\boldsymbol {r}_k$ and sum over all *k* particles. Doing so, and substituting $\boldsymbol {x}$ for a point $\boldsymbol {p}$ on the sphere of radius $r_{\rm t}$, hence $\boldsymbol {x}=r_{\rm t}\, \hat{\boldsymbol {p}}$, results in equations (5) and (6).

## APPENDIX C: ALTERNATIVE METHODS OF DETERMINING THE BAR FORMATION PATHWAY

In Figs C1 and C2, we show two alternative methods to separate bar formation episodes into ‘secular’ and ‘tidal’ bars – definitions which we have shown to be largely arbitrary in a cosmological context. However, for the sake of comparison, we discuss below whether arbitrary thresholds in our tidal strength parameter, $\mathcal {S}_{\rm bar}$, can agree with the results of previous methods.

In Fig. C1, we compare our results with the method used in Y. Rosas-Guevara et al. (2024), which follows the galaxy merger trees to determine if a merger occurs near the time of bar formation. Y. Rosas-Guevara et al. (2024) identify the time of the closest merger to $t_{\rm bar}$ along the main progenitor branch with a stellar mass ratio $\mu \ge 0.1$, where $\mu = M_{\star }^{(2)}/M_{\star }^{(1)}$, and $M_{\star }^{(1)}$ is the stellar mass of the more massive galaxy (see C. Bottrell et al. 2024, for details on characterizing the merger histories of TNG50 galaxies), and search for such mergers within $t_{\rm bar}\pm 2t_{\rm dyn}$. Galaxies which experience these mergers within this time window form ‘tidal bars’. They also search for flybys within $t_{\rm bar}\pm 2t_{\rm dyn}$ with mass ratio $\mu \ge 0.1$ that pass within $100\, {\rm kpc}$ of the bar-forming galaxies; these are also labelled as ‘tidal bars’. All the other bar formation events would be labelled as ‘secular bars’.

In the left panel of Fig. C1, we see how this definition compares against our selection on $f_{\star }(t_{\rm bar})$ used in this work, where we show histograms of the time of the nearest $\mu \ge 0.1$ merger (normalized by $t_{\rm dyn}$) for all galaxies in our sample as a thick black line, and the distribution of our $f_{\star }(t_{\rm bins})$ as coloured, hatched histograms. The shaded region indicates the window within which a merger must occur for the bar to be tidal by the Y. Rosas-Guevara et al. (2024) definition. A clear spike in mergers at $t_{\rm bar}$ indicates the importance of merger-driven tidal interactions in bar formation, but the long tail in the distribution also shows that many bars have $\mu \ge 0.1$ mergers far before $t_{\rm bar}$. There is an approximately equal number of bars in the different $f_{\star }(t_{\rm bar})$ bins that form in the same snapshot as a $\mu \ge 0.1$ merger, as expected from Fig. 5. However, there are far more highly stellar-dominated bars with nearest mergers far before $t_{\rm bar}$, and nearly no bar forming DM-dominated galaxies in the same range, indicating marginal agreement between our selections and the Y. Rosas-Guevara et al. (2024) definitions; this should be no surprise, as these definitions are not related.

A fairer comparison is shown in the right panel, where we show how three arbitrary $\mathcal {S}_{\rm bar}$ thresholds agree with the Y. Rosas-Guevara et al. (2020) definitions: all galaxies with $\mathcal {S}_{\rm bar} \ge 0.2$ form their bars within $\pm 2t_{\rm dyn}$ of a $\mu \ge 0.1$ merger. In contrast, far fewer bars with $\mathcal {S}_{\rm bar} < 0.1$ and $0.1 \le \mathcal {S}_{\rm bar} < 0.2$ form during this window. Ultimately, we choose not to use this method because there is no clear time window or $\mathcal {S}_{\rm bar}$ that can delineate tidal and secular bar formation, and there is no reason that $\mu \ge 0.1$ mergers *must* lead to bar formation, particularly if the tidal forces during the merger are particularly small once accounting for the rest of the nearby environment. Furthermore, the choice of $\mu \ge 0.1$ is highly arbitrary; M. K. Cavanagh et al. (2022) indicated that bar formation can be associated to mergers with $\mu$ far above and below this.

An alternative method uses the scaled tidal index, the scalar $\mathcal {T}$ (derived as part of equation 6, but see e.g. S. Ansar et al. 2025, for a recent application). $\mathcal {T}$ measures a simplified tidal force on the bar-forming galaxy individually for every satellite in the same halo as the bar-forming galaxy, for each snapshot within one halo $t_{\rm dyn}$ before $t_{\rm bar}$. When calculating the scalar $\mathcal {T}$, we choose $m = M_{\rm sat}$ and $r = D_{\rm sat}$, the total bound mass of any satellite and the distance between the centres of potential of our barred galaxies and the associated satellite, respectively. We again choose $r_{\rm t} = 4r_{\star , 1/2}$ as the outer radius enclosing the mass $M_{\rm t}(< r_{\rm t})$ of our barred galaxy.

In Fig. C2, we show how $\max (\mathcal {T})$ measured before $t_{\rm bar}$ compares to our $\mathcal {S}_{\rm bar}$ metric. Galaxies are coloured by our $f_{\star }(t_{\rm bar})$ selections, respectively. S. Ansar et al. (2025) and P. D. López et al. (2024) used $\max (\mathcal {T}) \gtrsim -2.45$ to divide the tidal and secular bars, in part because C. W. Purcell et al. (2011) showed that satellite interactions with this value of $\max (\mathcal {T})$ can induce a strong bar in a MW-mass galaxy; we show this as a horizontal dashed grey line. Bars in our sample form during a variety of $\max (\mathcal {T})$, but galaxies with $\mathcal {S}_{\rm bar} < 0.1$ are found at low values, and galaxies with $\mathcal {S}_{\rm bar} \ge 0.2$ are found at higher values, often above $\max (\mathcal {T}) = -2.45$. Some overlap exists in this parameter space, but primarily from galaxies with $0.1 \le \mathcal {S}_{\rm bar} < 0.2$, as expected. Our decision not to use $\max (\mathcal {T})$ is explained in Section 4.

## APPENDIX D: BAR PROPERTIES AT THE TIME OF BAR FORMATION

While we show the time evolution of normalised bar properties in Fig. 7, it can also be illuminating to view the unnormalized properties of bars at the time of bar formation, and at $z=0$. In Fig. D1, we show the bar strength, length, and pattern speed, all in physical units, as a function of $M_{\star }$ measured at $t=t_{\rm bar}$ and $z=0$ in the left and right columns, respectively. Galaxies are binned based on $f_{\star }(t_{\rm bar})$, and coloured as before. Thick lines of the same colour indicate sub-sample medians as a function of $M_{\star }$.

At the time of bar formation, bars that form in highly stellar-dominated discs (often through secular evolution), tend to be stronger, longer, and more quickly rotating at fixed mass when compared to bars that form in less stellar-dominated discs. Galaxies that form bars in DM-dominated discs (i.e. often tidal bars), are among the weakest, and most slowly rotating. However, these same galaxies are also particularly long, especially when found at stellar masses below $M_{\star } \le 10^{10}\, {{\rm M}_{\odot }}$.

Similarly, at $z=0$, some trends emerge: for instance, bars that formed in highly stellar-dominated discs tend to be strongest, and show relatively low pattern speeds, indicative of the slowdown associated with dynamical friction between the bar and DM halo (e.g. S. Tremaine & M. D. Weinberg 1984; M. D. Weinberg 1985; L. Hernquist & M. D. Weinberg 1992; E. Athanassoula 2007). On the other hand, galaxies that formed bars in DM-dominated systems are relatively weak and quickly rotating, possibly suggesting that the tidal interactions that frequently trigger bars in these galaxies can influence their subsequent evolution. The bar lengths are indistinguishable between galaxies with different $f_{\star }(t_{\rm bar})$, but have shown a general decline since $t=t_{\rm bar}$, as have bar pattern speeds. The bar strengths, on the other hand, have on average risen, and appear to be strongest in the lowest mass discs of our sample.

## References

[bib1] Algorry D. G. et al., 2017, MNRAS, 469, 1054 10.1093/mnras/stx1008

[bib2] Ansar S., Pearson S., Sanderson R. E., Arora A., Hopkins P. F., Wetzel A., Cunningham E. C., Quinn J., 2025, ApJ, 978, 37 10.3847/1538-4357/ad8b45

[bib3] Athanassoula E. , 2002, ApJ, 569, L83 10.1086/340784

[bib4] Athanassoula E. , 2003, MNRAS, 341, 1179 10.1046/j.1365-8711.2003.06473.x

[bib5] Athanassoula E. , 2007, MNRAS, 377, 1569 10.1111/j.1365-2966.2007.11711.x

[bib6] Athanassoula E. , 2013, in Falcón-Barroso J., Knapen J. H., eds, Secular Evolution of Galaxies. p. 305 Cambridge University Press Cambridge, UK

[bib7] Athanassoula E., Sellwood J. A., 1986, MNRAS, 221, 213 10.1093/mnras/221.2.213

[bib8] Athanassoula E., Machado R. E. G., Rodionov S. A., 2013, MNRAS, 429, 1949 10.1093/mnras/sts452

[bib9] Aumer M., Binney J., 2017, MNRAS, 470, 2113 10.1093/mnras/stx1300

[bib10] Berentzen I., Athanassoula E., Heller C. H., Fricke K. J., 2004, MNRAS, 347, 220 10.1111/j.1365-2966.2004.07198.x

[bib11] Bland-Hawthorn J., Tepper-Garcia T., Agertz O., Freeman K., 2023, ApJ, 947, 80 10.3847/1538-4357/acc469

[bib12] Bland-Hawthorn J., Tepper-Garcia T., Agertz O., Federrath C., 2024, ApJ, 968, 86 10.3847/1538-4357/ad4118

[bib13] Bottrell C. et al., 2024, MNRAS, 527, 6506 10.1093/mnras/stad2971

[bib14] Bournaud F., Combes F., Semelin B., 2005, MNRAS, 364, L18 10.1111/j.1745-3933.2005.00096.x

[bib15] Cavanagh M. K., Bekki K., Groves B. A., Pfeffer J., 2022, MNRAS, 510, 5164 10.1093/mnras/stab3786

[bib16] Combes F., Sanders R. H., 1981, A&A, 96, 164

[bib17] Conselice C. J. et al., 2024, MNRAS, 531, 4857 10.1093/mnras/stae1180

[bib18] Correa C. A., Schaye J., Clauwens B., Bower R. G., Crain R. A., Schaller M., Theuns T., Thob A. C. R., 2017, MNRAS, 472, L45 10.1093/mnrasl/slx133

[bib19] Curir A., Mazzei P., Murante G., 2006, A&A, 447, 453 10.1051/0004-6361:20053418

[bib20] Das M., Laurikainen E., Salo H., Buta R., 2008, Ap&SS, 317, 163 10.1007/s10509-008-9873-9

[bib21] de Sá-Freitas C. et al., 2023, A&A, 671, A8 10.1051/0004-6361/202244667

[bib22] de Sá-Freitas C. et al., 2025, A&A, 698, A5 10.1051/0004-6361/202453367

[bib23] Dehnen W., Semczuk M., Schönrich R., 2023, MNRAS, 518, 2712 10.1093/mnras/stac3184

[bib24] Dolag K., Borgani S., Murante G., Springel V., 2009, MNRAS, 399, 497 10.1111/j.1365-2966.2009.15034.x

[bib25] Dubinski J., Berentzen I., Shlosman I., 2009, ApJ, 697, 293 10.1088/0004-637X/697/1/293

[bib26] Efstathiou G., Lake G., Negroponte J., 1982, MNRAS, 199, 1069 10.1093/mnras/199.4.1069

[bib27] Elmegreen D. M., Sundin M., Elmegreen B., Sundelius B., 1991, A&A, 244, 52

[bib28] Emsellem E. et al., 2022, A&A, 659, A191 10.1051/0004-6361/202141727

[bib29] Espejo Salcedo J. M. et al., 2025, A&A, 700, A42 10.1051/0004-6361/202554725

[bib30] Ferreira L. et al., 2023, ApJ, 955, 94 10.3847/1538-4357/acec76

[bib31] Fragkoudi F., Di Matteo P., Haywood M., Gómez A., Combes F., Katz D., Semelin B., 2017, A&A, 606, A47 10.1051/0004-6361/201630244

[bib32] Fragkoudi F. et al., 2020, MNRAS, 494, 5936 10.1093/mnras/staa1104

[bib33] Fragkoudi F., Grand R. J. J., Pakmor R., Gómez F., Marinacci F., Springel V., 2025, MNRAS, 538, 1587 10.1093/mnras/staf389

[bib34] Frankel N. et al., 2022, ApJ, 940, 61

[bib35] Fraser-McKelvie A. et al., 2020, MNRAS, 499, 1116 10.1093/mnras/staa2866

[bib36] Fraser-McKelvie A. et al., 2025, A&A, 700, A237 10.1051/0004-6361/202452891

[bib37] Friedli D., Benz W., 1993, A&A, 268, 65

[bib38] Frosst M., Obreschkow D., Ludlow A., 2024, MNRAS, 534, 313 10.1093/mnras/stae2086

[bib39] Frosst M., Obreschkow D., Ludlow A., Bottrell C., Genel S., 2025, MNRAS, 537, 3543 10.1093/mnras/staf255

[bib40] Fujii M. S., Bédorf J., Baba J., Portegies Zwart S., 2018, MNRAS, 477, 1451 10.1093/mnras/sty711

[bib41] Gadotti D. A. et al., 2019, MNRAS, 482, 506 10.1093/mnras/sty2666

[bib42] Gargiulo I. D. et al., 2022, MNRAS, 512, 2537 10.1093/mnras/stac629

[bib43] Gavazzi G. et al., 2015, A&A, 580, A116 10.1051/0004-6361/201425351

[bib44] Ghosh S., Di Matteo P., 2024, A&A, 683, A100 10.1051/0004-6361/202347763

[bib45] Ghosh S., Fragkoudi F., Di Matteo P., Saha K., 2023, A&A, 674, A128 10.1051/0004-6361/202245275

[bib46] Gnedin O. Y. , 2003, ApJ, 582, 141 10.1086/344636

[bib47] Grady J., Belokurov V., Evans N. W., 2020, MNRAS, 492, 3128 10.1093/mnras/stz3617

[bib48] Guo R., Mao S., Athanassoula E., Li H., Ge J., Long R. J., Merrifield M., Masters K., 2019, MNRAS, 482, 1733 10.1093/mnras/sty2715

[bib49] Guo Y. et al., 2023, ApJ, 945, L10 10.3847/2041-8213/acacfb

[bib50] Guo Y. et al., 2025, ApJ, 985, 181 10.3847/1538-4357/adc8a7

[bib51] Hammer F., Flores H., Puech M., Yang Y. B., Athanassoula E., Rodrigues M., Delgado R., 2009, A&A, 507, 1313 10.1051/0004-6361/200912115

[bib52] Harris C. R. et al., 2020, Nature, 585, 357 10.1038/s41586-020-2649-2 32939066 PMC7759461

[bib53] Hernquist L., Weinberg M. D., 1992, ApJ, 400, 80 10.1086/171975

[bib54] Hohl F. , 1971, ApJ, 168, 343 10.1086/151091

[bib55] Hunter J. D. , 2007, Comput. Sci. Eng., 9, 90 10.1109/MCSE.2007.55

[bib56] Iles E. J., Pettitt A. R., Okamoto T., Kawata D., 2024, MNRAS, 527, 2799 10.1093/mnras/stad3377

[bib57] Izquierdo-Villalba D. et al., 2022, MNRAS. 514:1006 10.1093/mnras/stac1413

[bib58] Karachentsev I. D., Makarov D. I., 1999, in Barnes J. E., Sanders D. B., eds, IAU Symp. Vol. 186, Galaxy Interactions at Low and High Redshift. p. 109 Kyoto, Japan

[bib59] Khoperskov S., Minchev I., Steinmetz M., Ratcliffe B., Walcher J. C., Libeskind N. I., 2024, MNRAS, 533, 3975 10.1093/mnras/stae1902

[bib60] Kim T., Athanassoula E., Sheth K., Bosma A., Park M.-G., Lee Y. H., Ann H. B., 2021, ApJ, 922, 196 10.3847/1538-4357/ac2300

[bib61] Klypin A., Valenzuela O., Colín P., Quinn T., 2009, MNRAS, 398, 1027 10.1111/j.1365-2966.2009.15187.x

[bib62] Kraljic K., Bournaud F., Martig M., 2012, ApJ, 757, 60 10.1088/0004-637X/757/1/60

[bib63] Lang M., Holley-Bockelmann K., Sinha M., 2014, ApJ, 790, L33 10.1088/2041-8205/790/2/L33

[bib64] Le Conte Z. A. et al., 2024, MNRAS, 530, 1984 10.1093/mnras/stae921

[bib65] Łokas E. L. , 2018, ApJ, 857, 6 10.3847/1538-4357/aab4ff

[bib66] Łokas E. L., Athanassoula E., Debattista V. P., Valluri M., Pino A. d., Semczuk M., Gajda G., Kowalczyk K., 2014, MNRAS, 445, 1339 10.1093/mnras/stu1846

[bib67] Łokas E. L., Ebrová I., del Pino A., Sybilska A., Athanassoula E., Semczuk M., Gajda G., Fouquet S., 2016, ApJ, 826, 227 10.3847/0004-637X/826/2/227

[bib68] López P. D., Scannapieco C., Cora S. A., Gargiulo I. D., 2024, MNRAS, 529, 979 10.1093/mnras/stae576

[bib69] Lu S., Du M., Debattista V. P., 2025, A&A, 697, A236 10.1051/0004-6361/202453143

[bib70] Marinacci F. et al., 2018, MNRAS, 480, 5113 10.1093/mnras/sty2206

[bib71] Martinez-Valpuesta I., Aguerri J. A. L., González-García A. C., Dalla Vecchia C., Stringer M., 2017, MNRAS, 464, 1502 10.1093/mnras/stw2500

[bib72] Mayer L., Wadsley J., 2004, MNRAS, 347, 277 10.1111/j.1365-2966.2004.07202.x

[bib73] Melvin T. et al., 2014, MNRAS, 438, 2882 10.1093/mnras/stt2397

[bib74] Merrow A., Grand R. J. J., Fragkoudi F., Martig M., 2024, MNRAS, 531, 1520 10.1093/mnras/stae1250

[bib75] Miwa T., Noguchi M., 1998, ApJ, 499, 149 10.1086/305611

[bib76] Moetazedian R., Polyachenko E. V., Berczik P., Just A., 2017, A&A, 604, A75 10.1051/0004-6361/201630024

[bib77] Molaeinezhad A., Falcón-Barroso J., Martínez-Valpuesta I., Khosroshahi H. G., Vazdekis A., La Barbera F., Peletier R. F., Balcells M., 2017, MNRAS, 467, 353 10.1093/mnras/stx051

[bib78] Moore B., Lake G., Katz N., 1998, ApJ, 495, 139 10.1086/305264

[bib79] Naiman J. P. et al., 2018, MNRAS, 477, 1206 10.1093/mnras/sty618

[bib80] Nelson D. et al., 2018, MNRAS, 475, 624 10.1093/mnras/stx3040

[bib81] Nelson D. et al., 2019, Comput. Astrophys. Cosmol., 6, 2 10.1186/s40668-019-0028-x

[bib82] Noguchi M. , 1987, MNRAS, 228, 635 10.1093/mnras/228.3.635

[bib83] Oh S. H., Kim W.-T., Lee H. M., Kim J., 2008, ApJ, 683, 94 10.1086/588184

[bib84] Pearson S. et al., 2016, MNRAS, 459, 1827 10.1093/mnras/stw757

[bib85] Peschken N., Łokas E. L., 2019, MNRAS, 483, 2721 10.1093/mnras/sty3277

[bib86] Pettitt A. R., Wadsley J. W., 2018, MNRAS, 474, 5645 10.1093/mnras/stx3129

[bib87] Pillepich A. et al., 2018a, MNRAS, 473, 4077 10.1093/mnras/stx2656

[bib88] Pillepich A. et al., 2018b, MNRAS, 475, 648 10.1093/mnras/stx3112

[bib89] Pillepich A. et al., 2024, MNRAS, 535, 1721 10.1093/mnras/stae2165

[bib90] Planck Collaboration XIII , 2016, A&A, 594, A13 10.1051/0004-6361/201525830

[bib91] Purcell C. W., Bullock J. S., Tollerud E. J., Rocha M., Chakrabarti S., 2011, Nature, 477, 301 10.1038/nature10417 21921911

[bib92] Reddish J. et al., 2022, MNRAS, 512, 160 10.1093/mnras/stac494

[bib93] Rodriguez-Gomez V. et al., 2015, MNRAS, 449, 49 10.1093/mnras/stv264

[bib94] Rodriguez-Gomez V. et al., 2017, MNRAS, 467, 3083 10.1093/mnras/stx305

[bib95] Romano-Díaz E., Shlosman I., Heller C., Hoffman Y., 2008, ApJ, 687, L13 10.1086/593168

[bib96] Rosas-Guevara Y. et al., 2020, MNRAS, 491, 2547 10.1093/mnras/stz3180

[bib97] Rosas-Guevara Y. et al., 2022, MNRAS, 512, 5339 10.1093/mnras/stac816

[bib98] Rosas-Guevara Y., Bonoli S., Misa Moreira C., Izquierdo-Villalba D., 2024, A&A, 684, A179 10.1051/0004-6361/202349003

[bib99] Roshan M., Ghafourian N., Kashfi T., Banik I., Haslbauer M., Cuomo V., Famaey B., Kroupa P., 2021, MNRAS, 508, 926 10.1093/mnras/stab2553

[bib100] Sales L. V., Navarro J. F., Schaye J., Dalla Vecchia C., Springel V., Booth C. M., 2010, MNRAS, 409, 1541 10.1111/j.1365-2966.2010.17391.x

[bib101] Sanders J. L., Kawata D., Matsunaga N., Sormani M. C., Smith L. C., Minniti D., Gerhard O., 2024, MNRAS, 530, 2972 10.1093/mnras/stae711

[bib102] Scannapieco C., Athanassoula E., 2012, MNRAS, 425, L10 10.1111/j.1745-3933.2012.01291.x

[bib103] Sellwood J. A. , 2014, Rev. Mod. Phys., 86, 1 10.1103/RevModPhys.86.1

[bib104] Sellwood J. A., Carlberg R. G., 1984, ApJ, 282, 61 10.1086/162176

[bib105] Semczuk M., Łokas E. L., del Pino A., 2017, ApJ, 834, 7 10.3847/1538-4357/834/1/7

[bib106] Semczuk M., Dehnen W., Schönrich R., Athanassoula E., 2024, A&A, 692, A159 10.1051/0004-6361/202451521

[bib107] Springel V. , 2010, MNRAS, 401, 791 10.1111/j.1365-2966.2009.15715.x

[bib108] Springel V., White S. D. M., Tormen G., Kauffmann G., 2001, MNRAS, 328, 726 10.1046/j.1365-8711.2001.04912.x

[bib109] Springel V. et al., 2018, MNRAS, 475, 676 10.1093/mnras/stx3304

[bib110] Tremaine S., Weinberg M. D., 1984, ApJ, 282, L5 10.1086/184292

[bib111] van de Sande J., Fraser-McKelvie A., Fisher D. B., Martig M., Hayden M. R., Geckos Survey Collaboration, 2024 in Galactic Bars: Driving and Decoding Galaxy Evolution. p. 27 Tabatabaei F., Barbuy B., Ting T.-S., Proceedings of the International Astronomical Union 377:10.5281/zenodo.8131220

[bib112] Virtanen P. et al., 2020, Nat. Methods, 17, 261 10.1038/s41592-019-0686-2 32015543 PMC7056644

[bib113] Weinberg M. D. , 1985, MNRAS, 213, 451 10.1093/mnras/213.3.451

[bib114] Weinberger R. et al., 2017, MNRAS, 465, 3291 10.1093/mnras/stw2944

[bib115] Weisz D. R. et al., 2011, ApJ, 743, 8 10.1088/0004-637X/743/1/8

[bib116] Zana T., Dotti M., Capelo P. R., Bonoli S., Haardt F., Mayer L., Spinoso D., 2018a, MNRAS, 473, 2608 10.1093/mnras/stx2503

[bib117] Zana T., Dotti M., Capelo P. R., Mayer L., Haardt F., Shen S., Bonoli S., 2018b, MNRAS, 479, 5214 10.1093/mnras/sty1850

[bib118] Zana T., Capelo P. R., Dotti M., Mayer L., Lupi A., Haardt F., Bonoli S., Shen S., 2019, MNRAS, 488, 1864 10.1093/mnras/stz1834

[bib119] Zana T. et al., 2022, MNRAS, 515, 1524 10.1093/mnras/stac1708

[bib120] Zavala J., Okamoto T., Frenk C. S., 2008, MNRAS, 387, 364 10.1111/j.1365-2966.2008.13243.x

[bib121] Zheng Y., Shen J., 2025, ApJ, 979, 60 10.3847/1538-4357/ad9bae

